# Investigating Monoliths (Vinyl Azlactone-co-Ethylene Dimethacrylate) as a Support for Enzymes and Drugs, for Proteomics and Drug-Target Studies

**DOI:** 10.3389/fchem.2019.00835

**Published:** 2019-12-03

**Authors:** Christine Olsen, Frøydis Sved Skottvoll, Ole Kristian Brandtzaeg, Christian Schnaars, Pål Rongved, Elsa Lundanes, Steven Ray Wilson

**Affiliations:** ^1^Department of Chemistry, University of Oslo, Oslo, Norway; ^2^Department of Pharmaceutical Chemistry, University of Oslo, Oslo, Norway

**Keywords:** monolithic support, immobilized enzyme reactor, target deconvolution, drug-target interaction, immobilized drug reactor

## Abstract

Prior to mass spectrometry, on-line sample preparation can be beneficial to reduce manual steps, increase speed, and enable analysis of limited sample amounts. For example, bottom-up proteomics sample preparation and analysis can be accelerated by digesting proteins to peptides in an on-line enzyme reactor. We here focus on low-backpressure 100 μm inner diameter (ID) × 160 mm, 180 μm ID × 110 mm or 250 μm ID × 140 mm vinyl azlactone-co-ethylene dimethacrylate [poly(VDM-co-EDMA)] monoliths as supports for immobilizing of additional molecules (i.e., proteases or drugs), as the monolith was expected to have few unspecific interactions. For on-line protein digestion, monolith supports immobilized with trypsin enzyme were found to be suited, featuring the expected characteristics of the material, i.e., low backpressure and low carry-over. Serving as a functionalized sample loop, the monolith units were very simple to connect on-line with liquid chromatography. However, for on-line target deconvolution, the monolithic support immobilized with a Wnt pathway inhibitor was associated with numerous secondary interactions when exploring the possibility of selectively trapping target proteins by drug-target interactions. Our initial observations suggest that (poly(VDM-co-EDMA)) monoliths are promising for e.g., on-line bottom-up proteomics, but not a “fit-for-all” material. We also discuss issues related to the repeatability of monolith-preparations.

## Introduction

With the recent advances in liquid chromatography (LC) and mass spectrometry (MS), sample preparation is the most time-consuming part of the method and is often the largest contributor to false analysis results. Sample preparation may be separated into two main categories: off-line procedures and on-line procedures. In on-line sample preparation techniques, the samples are prepared and measured in the same workflow in a closed system, often offering improved performance as both loss of sample and possibility of contamination is reduced. In an automated on-line method the contribution from human error is reduced, increasing the repeatability/reproducibility of the method (Kataoka, 2003; Nováková and Vlčková, 2009; Pan et al., 2014).

A specific on-line sample preparation step that can be beneficial to have up-stream in an LC-MS analysis system, is the digestion of proteins to peptides for bottom-up proteomics. This can be achieved by immobilizing enzymes on suitable supports compatible with a capillary- or nanoLC-MS set-up, e.g., particle packed (Moore et al., 2016), porous layer open tubular (PLOT) (Brandtzaeg et al., 2017) and monolithic capillaries (Geiser et al., 2008).

The particle packed variant can be associated with high backpressure due to the small particle dimensions (1.5 to 5 μm), dense packing and narrow pores (10 to 30 nm/100 to 300 Å). The high pressure force the liquid to flow around the particles instead of into the pores, reducing the surface of active sites (e.g., the immobilized enzyme) available to interact with the proteins (Xie et al., 1999). In addition, high backpressure can require complex solutions for introducing samples to the enzyme reactor. Also, packed columns often contain filters, or frits, in the ends of the capillary for keeping the particles in place, which increases dead-volumes in the connections that can be especially evident when operating miniaturized systems.

The PLOT and the capillary monolithic formats do not need frits as the porous structure is covalently attached to the wall as a thin layer or a rigid porous structure that fills the entire cavity, respectively (Eeltink et al., 2017). With the same length and ID, monolithic columns offer a larger available surface area than PLOT columns and at a much lower backpressure compared to particle packed columns (Platonova and Tennikova, 2005; Geiser et al., 2008). Another format of PLOT capillaries that can provide an increased number of active sites by increasing the surface area are multichannel columns (i.e., a single piece of capillary with several channels). We have successfully applied multichannel columns for enzyme digestion in an on-line LC-MS system for detection of ricin (Brandtzaeg et al., 2017). However, multichannel PLOT formats can (today) be quite expensive to produce, due to their custom housings, and are less accessible compared to traditional capillaries.

Monoliths can be silica-based or organic polymer-based, where organic polymer monoliths are typically regarded as more suited for macromolecules (Masini and Svec, 2017). Organic polymer monoliths will be the focus of this study as they may have the highest potential for immobilization of ligands due to their characteristics of low backpressure, stability in most solvents and in a wide pH range, accessibility of the active sites due to their pore sizes and structure, and the possibilities of tailoring the functionality of the polymer (Svec, 2006; Krenkova and Svec, 2009; Vlakh and Tennikova, 2013; Safdar et al., 2014; Meller et al., 2017; Naldi et al., 2018). The monolithic format used in this study is also quite inexpensive as no custom parts and reagents are used. The organic monolith immobilized enzyme reactor (IMER) should also be very straightforward to couple on-line with separation systems, e.g., as a functionalized injection loop. To our knowledge, this approach has not been employed for monolith reactors, and is applied in this paper.

There exists a wide variety of organic polymer monoliths which characteristics depend on the monomers used for polymerization (Svec, 2010). The selected vinyl azlactone-co-ethylene dimethacrylate = (poly(VDM-co-EDMA)) monolith is a polymer formed by a relatively polar and a reactive monomer, EDMA and VDM, respectively, and the resulting rather hydrophilic surface will prevent contribution of non-specific hydrophobic interactions with proteins and peptides. The following ring-opening reaction between VDM and a functional group (e.g., amino, hydroxyl, and thiol) will allow for post-modification of the monolithic surface for attachment of ligands, e.g., enzymes and drugs (Coleman et al., 1990; Platonova and Tennikova, 2005). The hydrophilic surface and the post-modification of poly(VDM-co-EDMA) monoliths offer a potential versatile on-line support for different protein sample preparation methods depending on the nature of the immobilized ligands. Thus, in addition to investigating the poly(VDM-co-EDMA) monolith as a support for immobilization of a protease (i.e., trypsin), an IMER, the monolith was also immobilized with a modified Wnt-inhibitor drug, called here a *capti remedium ad monolitus* (CRAM) reactor (“monolith trapped drug”), as a possible tool for target deconvolution in drug discovery. The CRAM reactor would be used to trap the drug target through drug-target interactions, and subsequently elute purified target eluate for identification. The Wnt-inhibitor anti-cancer drug (LDW639) targeting tankyrase 1 and 2 (TNKS1/2) in the Wnt/β-catenin signaling pathway was selected as the model system for the CRAM reactor (Zhan et al., 2017). The inhibition of TNKS1/2 (Solberg et al., 2018) and an inactive Wnt/β-catenin signaling pathway (Mook et al., 2017; Nusse and Clevers, 2017) are attractive for treatment of several types of cancer.

## Experimental Section

The overview of chemicals used in the following experiments are presented in Supplementary Material Sections 1, 3, 4. The poly(VDM-co-EDMA) monoliths were prepared in polyimide-coated fused silica tubing with an 180 ± 6 or 250 ± 6 μm ID, both with an outer diameter (OD) of 360 ± 6 μm, from Polymicro Technologies now a part of Molex (Lisle, IL, USA). The monolithic polymer support for immobilization of enzymes and drugs was formed *in-situ* by free-radical addition polymerization of EDMA and VDM utilizing α-α'-azoisobutyronitrile (AIBN) as initiator. In brief, the fused silica capillaries were filled with 1 M NaOH using an previously described in-house pressurized filling system and sealed in both ends by septa (Berg et al., 2017). After 22 h, the capillaries were washed with water and ACN before being dried with N_2_(g). The NaOH treated capillaries were filled with a silanization solution [0.5% 2,2-diphenyl-1-picrylhydrazyl (DPPH), 66.08% *N, N*-dimethylformamide (DMF) and 32.32% 3-(trimethoxysilyl)propyl methacrylate (ɤ-MAPS), (w/w/w)], which was sonicated for 5 min before filling. The filled capillaries were sealed by septa and placed in an oven (Shimadzu, Kyoto, Japan) at 110°C for 6 h. Subsequently, the capillaries were flushed with acetonitrile (ACN) and dried with N_2_(g). The silanization procedure was based on Hustoft et al. (2013). Finally, the capillaries were filled with a polymerization mixture [1% AIBN, 23% VDM, 16% EDMA, 34% 1-propanol and 26% 1,4-butanediol, (w/w/w/w/w)], sealed and placed in an oven at 70°C for 24 h. Subsequently, the capillaries were washed with acetone and dried with N_2_(g). The polymerization procedure was based on Geiser et al. (2008). The chemicals used for production of the poly(VDM-co-EDMA) monoliths were analyzed by proton nuclear magnetic resonance (^1^H-NMR), further details are given in Supplementary Material Section 6.

For characterization of the morphology of the monoliths, a micrograph of the cross-section was captured with a Quanta 200 FEG-E scanning electron microscope (SEM) from FEI Company (Hillsboro, OR, USA) now a part of Thermo Fisher Scientific. From the dry poly(VDM-co-EDMA) monolith, 1 cm was cut off and glued in an upright position on a sample holder with carbon tape. The sample holder was placed in the sample chamber before the chamber was pumped to low vacuum. A large field detector operating at 15.0 kV, 12 mm distance for the sample and with a 4.0 spot size was used to capture the micrographs.

For immobilizing trypsin to VDM on the monolithic support, a solution consisting of 0.25 mg/mL trypsin and 2.25 mg/mL benzamidine in 50 mM phosphate buffer (pH 7.2) was flushed through the monoliths for 3.5 h. The resulting immobilized enzyme reactors (IMERs) were filled with 50 mM ammonium acetate buffer (pH 8.75), sealed with septa and stored at 4°C.

**Figure 1:**
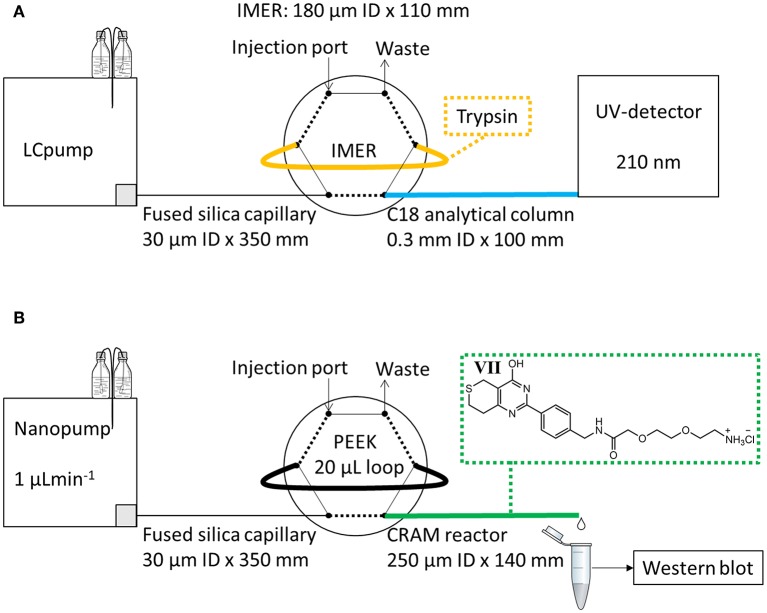
Experimental set-up for evaluation of **(A)** IMERs with trypsin and **(B)** CRAM reactors with modified LDW639 (**VII**).

The modified LDW639 Wnt-inhibitor drug was synthesized in-house from methyl-4-oxotetrahydro-2H-thiopyran-3-carboxylate (**I**, beta-keto ester, 95%) and 4-boc-aminomethylbenzamidine (**II**, boc-benzamidine, 97%), and modified by the addition of a linker (**V**, 2,2-dimethyl-4-oxo-3,8,11-trioxa-5-azatridecan-13-oic acid, 97%) all purchased from Fluorochem (Hadfield, United Kingdom). The finalized product (**VII**), structure shown in Figure 1, was examined for Wnt-signaling activity using a SuperTOPFlash-luciferase assay (STF-Luc) at the Unit of Cell Signaling, Oslo University Hospital. The synthesis, characterization and determination of Wnt-activity of modified LDW639 (**VII**) is described in Supplementary Material Section 3, Figures S4–S9.

The CRAM reactor was prepared by immobilizing the modified LDW639 Wnt-inhibitor drug on the poly(VDM-co-EDMA) monolith by flushing a solution of 5 mg/mL drug in 50 mM phosphate buffer (pH 7.2) through the capillary for 3 h (~0.5 mL). Additionally, a reference monolith was made by flushing 1 M monoethanolamine (MEA) through a monolith in the same manner. Both the CRAM reactor and the MEA monolith were filled with 50 mM phosphate buffer, sealed with septa and stored at 4°C.

For evaluation of the protein digestion potential of the immobilized enzyme reactors, solutions of 500 μg/mL reduced [by dithiothreitiol (DDT)] and alkylated [by iodoacetamide (IAM)] myoglobin dissolved in 50 mM ammonium acetate were used. The in-solution digested myoglobin solutions were prepared by adding 10 μg trypsin per 500 μg myoglobin, and incubation at 37°C for 45 min. The solutions were stored at −20°C.

To investigate the possibility of trapping the protein target of the immobilized Wnt-inhibitor, human embryonic kidney 293 (HEK293) cells were received, after cell cultivation, from the unit of Cell Signaling, Oslo University Hospital. The cell line HEK293 were purchased from American Type Culture Collection (ATCC, Manassas, VA, USA), and maintained according to the ATCC guidelines. For cell lysis with a non-denaturing buffer (4.0 mL glycerol, 4 mL 10x protease inhibitor, 700 mg sodium chloride, 28 mg imidazole, 3 mg DTT and 1.2 mL 1 M tris buffer (pH 8.0), diluted with water to 40 mL), a procedure based on Voronkov et al. (2013) was used. In brief, the cell samples (~1 million cells) were added 200 μL of the buffer and vortexed by pipetting up and down a minimum of 10 times. The samples were ultrasonicated at 40 kHz for 30 s before 15 min incubation on ice, and the ultrasonication step was repeated once before another 15 min of incubation on ice. After incubation, the samples were centrifuged for 15 min at 14,000 relative centrifugal force (rcf), and the supernatant was pipetted into new tubes and stored at −20°C.

For evaluation of protein digestion by the IMERs, an on-line IMER-LC-UV system utilizing an Agilent 1,100 series pump (Agilent technologies, Santa Clara, CA, USA) (LC pump in Figure 1A) connected to a two-position 6-port valve from Vici Valco (Houston, TX, USA) was used. The mobile phase reservoir A contained 0.1% trifluoroacetic acid (TFA)/ACN (95/5, v/v) while B contained 0.1% TFA in ACN. A 180 μm ID × 110 mm IMER was directly attached to two ports of the external valve as shown in Figure 1A. The porosity of the IMER was estimated to be 74% (by elution of a non-retained compound on a 250 μm ID × 141 mm poly(VDM-co-EDMA) compared to that on an empty 250 μm ID × 141 mm fused silica capillary), the volume of the reactor was 2 μL. A total of 10 μL of myoglobin solution was applied by syringe on to the 2 μL IMER injection loop, and was trapped in the loop for 5 min at room temperature. After digestion, the treated solution was transferred in 1 min to an in-house packed 0.3 × 100 mm BetaMax Neutral C18 (5 μm particle diameter) steel capillary analytical column for separation at a flow rate of 2 μLmin^−1^. A gradient was run at a flow rate of 10 μLmin^−1^ for 25 min bypassing the IMER (started after 1 min); at 0% B for 0–1.5 min, linearly increased to 55% B for from 1.5 to 17 min, kept at 55% B at 17–23 min, quickly increased to 90% B for 1 min and then reversed to 0% B for 1 min. Detection was performed at 210 nm by a WellChrome K-2600 UV-detector (Knauer, Berlin, Germany), equipped with a 65 nL (100 μm ID/375 μm OD) flow cell. A Perkin Elmer Nelson 900 series analog-to-digital interface (Waltham, MA, USA) and a computer with a TotalChrom software were used for obtaining the chromatograms.

An EASY nLC pump from Proxeon now a part of Thermo Fisher (nanopump in Figure 1B) with mobile phase reservoirs A and B both containing 100% HPLC-grade water was used for evaluation of trapping of TNKS1/2 on CRAM reactors and MEA monoliths. Injection was manually performed with a glass syringe using an external two-position 6-port valve from Vici Valco with an attached 20 μL polyetheretherketone (PEEK) sample loop from Proxeon. A 250 μm ID × 140 mm CRAM reactor was attached to the external port as shown in Figure 1B, and the pump was used at 1 μLmin^−1^ flow rate. From an estimate of 74% porosity the volume of the CRAM reactor was 5.1 μL. The CRAM reactor was first rinsed for 30 min with HPLC water, while the loop was manually loaded with 20 μL of cell lysate sample. For the next 30 min the sample was transported from the loop onto the CRAM reactor, and the eluate was collected in a tube marked “Flush 1.” During the last minute of collection, the valve was switched to load position and another 20 μL of sample was loaded onto the loop. For another 30 min the second portion of sample was transferred onto the CRAM reactor, and the eluate was collected in a tube marked “Flush 2.” For the subsequent 30 min, HPLC water was flushed through the loop and the CRAM reactor, and collected in a tube marked “Wash 1.” A second wash eluate was collected, while bypassing the loop, in a tube marked “Wash 2.” For elution of bound TNKS1/2 on the CRAM reactor, 20 μL of 2% formic acid in HPLC-grade water was applied on the loop, flushed through the reactor for 30 min (total volume 30 μL) and collected in a tube marked “EluateF,” which was added 5 μL of 1 M NaOH prior to and 5 μL following sample collection to immediately neutralize the sample. A second elution was performed in the same manner with 2% sodium dodecyl sulfate (SDS, denaturing agent) in 60 mM tris buffer (w/v) and collected in a tube marked “EluateS.” All of the collected eluates were analyzed by western blot for TNKS1/2 using actin as loading control. Western blot procedure is explained in detail in Supplementary Materials Section 4.

## Results and Discussion

In this project, an poly(VDM-co-EDMA) monolith was assessed as a support for immobilized ligands to enable on-line sample preparations in bottom-up proteomics and target deconvolution in drug discovery using trypsin and modified LDW639, respectively.

### IMERs: Trypsin Immobilized on Poly(VDM-co-EDMA) Monoliths for Digestion of Myoglobin

**Figure 2:**
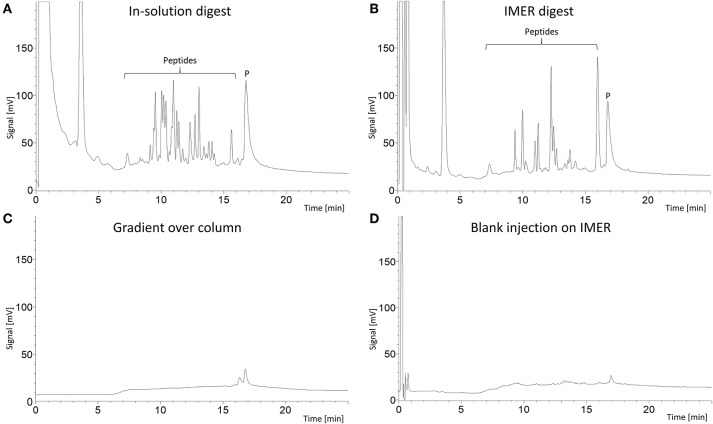
LC-UV chromatograms (210 nm) of: **(A)** 1.4 μL 500 μg/mL myoglobin (reduced and alkylated by DTT and IAM) in-solution digest (trypsin:protein, 1:50) injected into a 180 μm × 110 mm poly(VDM-co-EDMA) monolithic support. **(B)** 1.4 μL 500 μg/mL myoglobin (reduced and alkylated by DTT and IAM) injected into a 180 μm × 110 mm poly(VDM-co-EDMA) trypsin IMER for 5 min on-line digestion. **(C)** blank gradient run excluding the IMER and **(D)** injection of 1.4 μL 50 mM ammonium acetate on the 180 μm × 110 mm poly(VDM-co-EDMA) trypsin IMER which had been washed with 5 μL 30% ACN in 50 mM ammonium acetate following the myoglobin injection. The intact protein peak (P) occurs at 17 min, while the peptide peaks are concentrated from 7 to 16 min. The analytical column was a 0.3 × 100 mm BetaMax Neutral C18 (5 μm particle diameter) in a steel housing. Mobile phase A consisted of ACN/0.1% TFA (5/95, v/v), while mobile phase B consisted of 0.1% TFA in ACN. The gradient was performed with %B: at 0% for 0–1.5 min, linearly increased to 55% for from 1.5 to 17 min, kept at 55% at 17–23 min, quickly increased to 90% for 1 min and then reversed to 0% for 1 min.

First, we wanted to investigate if the poly(VDM-co-EDMA) monolith was suitable as a support for immobilized trypsin to enable fast on-line digestion of proteins to peptides. An evaluation of 9 replicates of 100 μm ID × 160 mm and 9 replicates of 180 μm ID × 110 mm poly(VDM-co-EDMA) monoliths were successfully prepared with a homogeneous, uniform morphology, shown in Supplementary Materials Section 5, Figures S11, S12, respectively. Important for practical use, the monoliths allowed easy manual injection using a loop on a 6-port valve due to low pressure (Figure 1). The poly(VDM-co-EDMA) monoliths show an average backpressure of 30 bar/m at a flow rate of 1 μL/min of 100% ACN with a permeability of 5.0 × 10^−14^ m^2^, calculated as described in Meller et al. (2016), based on backpressure measurements (n = 17). The permeability of the poly(VDM-co-EDMA) monolith is in same range as other organic monoliths (Vlakh et al., 2013; Volokitina et al., 2017), commercial particle packed columns (Song et al., 2014), and silica based monoliths (Motokawa et al., 2002; Zhang et al., 2013). The performance of the produced trypsin IMERs was evaluated by comparing the peptide fingerprint region of an on-line IMER digestion (5 min, Figure 2B) to that of myoglobin digested in-solution (Figure 2A). The peptide region confirmed that the IMERs had trypsin activity leading to digestion of myoglobin. To be able to compare the IMER digest with the in-solution digest, the in-solution digestion was injected on a monolithic support with no immobilized enzyme and analysis was run in the same manner as the IMER digestion. Possible carry-over of proteins and peptides was checked bypassing the IMER. The analytical column was responsible for the contribution of carry-over of protein as shown in Figure 2C. A blank run of 50 mM ammonium acetate following digestion of myoglobin on the IMER was executed after a simple washing step (5 μL of 30% ACN in 50 mM ammonium acetate). The simple washing step eliminated any significant carry-over from the IMER shown in Figure 2D. All 9 replicates of 100 μm ID IMERs and 9 replicates of 180 μm ID IMERs were successful in digesting myoglobin, as shown in Supplementary Materials Section 2, Figures S1, S2, respectively. On-line IMER-LC-MS was demonstrated with one reactor (Supplementary Materials Section 2, Figure S3); proteins spanning 10–70 kDa in mixture were readily identified with sequence coverages ranging from 26 to 69%. These values are comparable to that obtained with open tubular variants which included both trypsin and Lys C enzymes (Hustoft et al., 2014). However, we were unable to identify larger proteins e.g., fibrinogen alpha and transferrin, suggesting a protein size limitation regarding the current set-up.

Thus, the poly(VDM-co-EDMA) based trypsin IMERs allow easy manual injection due to low backpressure (no need for a separate loading pump, or e.g., having to time when digested fraction would enter the LC-system) and a simple washing step eliminates possible carry-over after successful digestion of myoglobin in 5 min. The trypsin poly(VDM-co-EDMA) IMERs can be used up-stream LC-MS in bottom-up proteomic studies to reduce time consumption on sample preparation and loss of sample.

### CRAM Reactors: LDW639 Immobilized Poly(VDM-co-EDMA) Monoliths for Selective Trapping and Elution of Target TNKS1/2

In drug discovery, development and optimization of new (and old) drugs depends on target identification (Terstappen et al., 2007), and a multitude of chemical proteomics methods exists (Kubota et al., 2019). However, target purification up-stream LC-MS would reduce loss of target and contaminations, give higher throughput and possible enable identification of low abundant targets.

An evaluation of poly(VDM-co-EDMA) monoliths immobilized with modified LDW639 drug for trapping and purification of target tankyrase 1/2 was executed. The modified LDW639 drug was successfully synthesized and found to inhibit Wnt-signaling in STF-Luc assay (Supplementary Materials Section 3, Figure S9). Both the 250 μm ID poly(VDM-co-EDMA) monolith immobilized with modified Wnt-pathway inhibitor (CRAM reactor) and the reference 250 μm ID poly(VDM-co-EDMA) monolith immobilized with monoethanolamine (MEA monolith) were successfully prepared; see below for discussion on the use of larger IDs than with the IMERs. A comparison between the CRAM reactor and the MEA monolith was carried out to assess whether or not immobilization of LDW639 offered trapping potential of the drug target TNKS1/2 in a different manner than the MEA monolith. Residues of the reactive VDM monomer in the CRAM reactor can give formation of covalent bonds between proteins and VDM (Coleman et al., 1990). Possible reaction between proteins and VDM was addressed by making the MEA monolith (described in Experimental), where all of the azlactone functionalities has been quenched by MEA, making the MEA monolith a suitable reference for no reaction between proteins and VDM.

**Figure 3:**
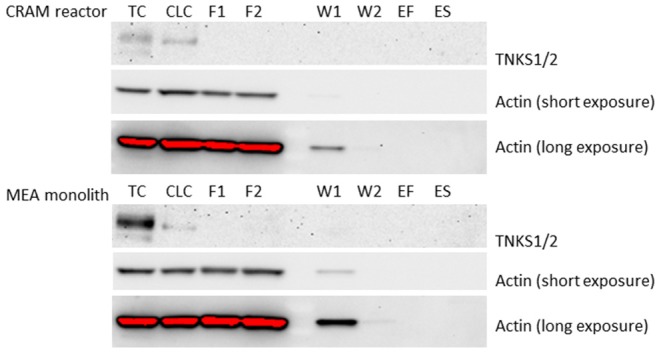
Western blot of: (TC–Tankyrase Control) lane of 21 μg of protein from 007-LK control and (CLC–Cell lysate control) lane of 36.3 μg of protein from cell lysate of HEK293. Samples collected from the CRAM reactor (Upper) and MEA monolith (Lower) in the following lanes: (F1) Flush of 36.3 μg protein from cell lysate of HEK293, (F2) Flush of 36.3 μg protein from cell lysate of HEK293, (W1) Wash 1 with water, (W2) Wash 2 with water, (ES) eluted with 2% formic acid and (ES) eluted with 2% SDS. The exposure time was 7,200 s for TNKS1/2, and for actin 10 s (short exposure) and 180 s (long exposure). The raw files from western blot are given in Supplementary material section 4, Figure S10.

The eluates collected from the CRAM reactor and the MEA monolith after applying HEK293 cell lysate were analyzed by western blot for target TNKS1/2 using actin as loading control, because actin is commonly expressed in all eukaryotic cell types. TNKS1/2 was not found in any eluates collected during flushing, washing or eluting from the CRAM reactor or the MEA monolith (Figure 3). TNKS1/2 was however present in a detectable amount in an equivalent aliquot of cell lysate as used for the CRAM reactor and the MEA monolith. TNKS1/2 was also detected in a positive TNKS1/2 control (cell lysate of HEK293 cells treated with Wnt-inhibitor 007-LK). The loading control actin was detectable in all controls and in the following eluates: Flush1, Flush2, Wash1 and Wash2, collected from both the CRAM reactor and the MEA monolith. Hence, TNKS1/2 was present in the cell lysate that was applied on the CRAM reactor and the MEA monolith, but TNKS1/2 was not eluted off in a western blot detectable amount during flushing with cell lysate, washing with water or eluting with acid or SDS. Thus, TNKS1/2 is retained, likely due to secondary interactions, on the poly(VDM-co-EDMA) based reactors which negates the selective affinity between drug and target in this format. The conditions attempted for elution of TNKS1/2 was changes in pH (2% formic acid, 1M and 3M NaOH), different ranges of salt (50 mM to 1 M ammonium acetate at pH 7.2), different percentages of ACN (10% to 50%) and denaturing agent (2% SDS). The induced changes in pH and salt concentration, and elution solutions consisting of ACN and SDS are common eluting methods for purification by affinity (Cuatrecasas et al., 1968; Shimizu et al., 2000). None of the selected elution solutions were successful at eluting TNKS1/2 in western blot detectable amount (only results for FA and SDS are shown).

The CRAM reactor used in this study shows that common elution conditions in affinity purification is not sufficient for eluting trapped target proteins from immobilized drugs. However, elution with 2% FA was sufficient for elution of peptides from poly(VDM-co-EDMA) monoliths with immobilized antibodies (Levernæs et al., 2018). The MEA monolith, with quenched azlactone functionalities, did not elute the target proteins, TNKS1/2, indicating that proteins are retained on the poly(VDM-co-EDMA) monolith by other interactions besides reaction with VDM. The unspecific interactions between proteins and the poly(VDM-co-EDMA) based CRAM reactors and MEA monoliths may indicated that an even more hydrophilic surface on the organic polymer is needed for intact protein assessments.

### Robustness of Recipe for Poly(VDM-co-EDMA) Monoliths Prepared in Capillaries

**Figure 4:**
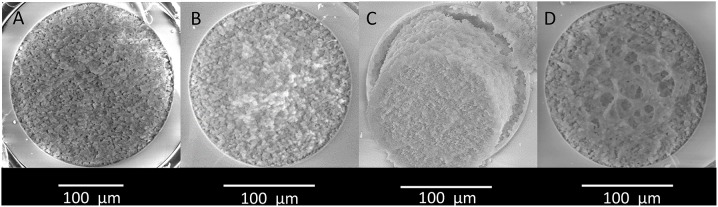
Micrographs of the cross-section of **(A)** a 250 μm ID and **(B)** a 180 μm ID poly(VDM-co-EDMA) monolith. **(C)** A 180 μm ID monolith not attached to the wall, and **(D)** a 180 μm ID monolith with large pores. The micrographs were captured by a large field detector (LFD) at 15.0 kV working at a distance of minimum 12 mm from the sample in low vacuum with a spot size of 4.0.

For immobilization of modified LDW639, monolithic supports with the highest possible amounts of active sites were desired. Thus, in addition to preparing the poly(VDM-co-EDMA) monolith in 180 μm ID capillaries, the monolith was also prepared in 250 μm ID capillaries. At this stage of the project, the uniform morphology was only successful in 250 μm ID capillaries (Figure 4A and Supplementary Materials Section 5, Figure S14). In contrast to the earlier production of monoliths for IMERs, the 180 μm ID capillaries later on displayed large pores disrupting the monolithic structure and was not correctly attached to the wall of the fused silica capillary (Figure 4 and Supplementary Materials Section 5, Figure S13). Because of this, the liquid chemicals used in production of the monoliths: DMF, γ-MAPS, EDMA, VDM, 1-propanol and 1,4-butanediol were replaced, and ^1^H-NMR spectra of the new and old chemicals were compared (Figures S15–S20 in Supplementary Materials Section 6). Virtually identical NMR spectra were obtained for the old and the new chemicals, indicating that no detectable degradation or contamination of the chemicals could explain why the poly(VDM-co-EDMA) monolith could not be produced in 180 μm ID capillaries anymore. However, monoliths prepared in 250 μm ID capillaries with new and old chemicals were not significantly different at a 95% confidence level based on backpressure measurements at 6 different flow rates.

Hence, we could not trace repeatability issues to the chemicals employed, suggesting that other conditions/factors e.g., subtle variations in humidity and temperature may play more substantial roles for the morphology of the monoliths (for example, the porogenic mixture consisting of 1-propanol and 1,4-butanediol is highly sensitive to presence of water). One of the reviewers suggested that including a protonation step of the silanols, using HCl after treatment of NaOH, may give a better activation of the silica surface before silanization by γ-MAPS and consequently a more consistent attachment of the monolith. Concerning the polymerization, the solution of the monomer EDMA consisted of 90–110 ppm of monomethyl ether hydroquinone (MeHQ, polymerization inhibitor). The MeQH has been suggested to be removed prior to polymerization to increase reproducible morphology of the monoliths.

The poly(VDM-co-EDMA) monolith has a good mechanical strength suitable for attachment as a loop on a 6-port valve and is easily prepared in fused silica capillaries with various IDs. The reactive VDM monomer also allows for tailoring toward specific applications by post-modification with ligands after formation of the organic polymer.

## Conclusion

The poly(VDM-co-EDMA) monoliths shows potential as trypsin-based IMERs with low backpressure and fast digestion of protein standards, and was very simple to incorporate in an on-line system as a functionalized loop with little carry-over. A next step will be to investigate their potential in on-line systems to be used in e.g., the field of “organ-on-a-chip” mass spectrometry-based proteomics. We have undertaken initial steps toward on-line drug/target studies using monolithic supports. Based on the same type of poly(VDM-co-EDMA) monolith, the CRAM reactor revealed that selective trapping and subsequent purified elution of high molecular weight protein targets (>110 kDa) in complex cell lysate was not straightforward, highlighting some limitations of this otherwise promising material and format. Nonetheless, due to the advantages that on-line drug/target studies can have, we are encouraged to explore alternative monoliths due to the format's versatility and ease of coupling with analytical instrumentation.

## Data Availability Statement

Datasets generated for this study are included in the article/Supplementary Material, with the exception of backpressure measurements. Requests to access the backpressure measurements data should be directed to the corresponding author.

## Author Contributions

CO, FS, and OB contributed to monolith work. CO performed LC-UV and LC-MS experiments. CO and FS performed biochemical experiments. CO, PR, and CS performed drug synthesis. SW, EL, and PR were supervisors. All authors contributed to planning experiments and writing the manuscript.

### Conflict of Interest

The authors declare that the research was conducted in the absence of any commercial or financial relationships that could be construed as a potential conflict of interest.
