# Supplementary material for: Investigating Monoliths (Vinyl Azlactone-co-Ethylene Dimethacrylate) as a Support for Enzymes and Drugs, for Proteomics and Drug-Target Studies
Source: Front Chem. 2019 Dec 3;7:835. doi: 10.3389/fchem.2019.00835 (PMC6902630; doi:10.3389/fchem.2019.00835)
Supplement: Supplementary file 1 [file Table_1.docx]

Supplementary Material for: Investigating monoliths (vinyl azlactone-co-ethylene dimethacrylate) as a support for enzymes and drugs, for proteomics and drug-target studies

Christine Olsen^1^, Frøydis Sved Skottvoll^1^, Ole Kristian Brandtzæg^1^, Christian Schnaars^2^, Pål Rongved^2^, Elsa Lundanes^1^, Steven Ray Wilson^1*^

^1^ University of Oslo, Department of Chemistry, Blindern, Oslo, Norway
^2^ University of Oslo, Department of Pharmaceutical Chemistry, Blindern, Oslo, Norway

*** Correspondence:**Steven Ray Wilson
[s.r.h.wilson@kjemi.uio.no](mailto:s.r.h.wilson@kjemi.uio.no)

# Chemicals

The reagents needed to produce the monolithic supports; α-α’-azoisobutyronitrile (AIBN, ≥ 98.0%), 1,4-butanediol (≥ 99%), *N*,*N*-dimethylformamide (DMF, 99.8%), 2,2-diphenyl-1-picrylhydrazyl, ethanolamine hydrochloride (DPPH, ≥ 99.0%), EDMA (98%), 1-propanol (99.7%), monobasic sodium phosphate (≥ 99.0%), 3-(trimethoxysilyl)propyl methacrylate (γ-MAPS, 98%), ethanolamine hydrochloride (≥ 99.0%), myoglobin from equine heart (≥ 90%), benzamidine (≥ 95.0 %), and trypsin (bovine pancreas, TPCK Treated, essentially salt-free, lyophilized powder, ≥ 10,000 BAEE units/mg protein) were all obtained from Sigma Aldrich (St. Louis, MO, USA). VDM (4,4-dimethyl-2-vinyl-2-oxazolin-5-one, ≥ 95%) was purchased from Polyscience Inc. (Warrington, PA, USA). Acetone (GPR rectapure) and acetonitrile (ACN, HPLC grade) were obtained from VWR (Radnor, PA, USA). Sodium hydroxide pellets (99.0%) and ammonium acetate (≥ 96%) were acquired from Merck (Darmstadt, Germany). Nitrogen gas (99.99%) was purchased from Praxair (Oslo, Norway).

Chemicals used for cell lysis and protein digestion: DL-dithiothreitol (DTT, Bioextra, ≥ 99.0%), imidazole (ACS reagent, ≥ 99%), iodoacetamide (IAM, BioUltra, ≥ 99%), glyserol (≥ 99%), urea (98%), ammonium bicarbonate (ABC, ReagentPlus®, ≥ 99.0%), SigmaFast™ protease inhibitor (general use), and formic acid (FA, reagent grade, ≥ 95%) were purchased from Sigma Aldrich. Sodium chloride (ACS reagent, ≥ 99.5%) was obtained from Merck. Tris buffer (1M, pH 8.0) was acquired from the Department of Microbiology at Oslo University Hospital.

The water used in the project was either HPLC grade water from VWR or type 1 water acquired from a Milli-Q® Integral water purification system equipped with a Q-POD dispenser (0.22 μm filter) from Merck Millipore (Billercia, MA, USA).

# Myoglobin digested on-line on poly(VDM-co-EDMA) trypsin IMERs

Digestion of 500 µg/µL myoglobin on 9 replicates of 100 µm ID x 160 mm and 180 µm ID x 110 mm poly(VDM-co-EDMA) trypsin IMERs is presented in **Figure S1** and **Figure S2**, respectively. The EDMA-co-VDM monoliths are marked with a code representing: Project (1=IMER or 2=CRAM), polymerization solution batch (A, B, C...), and replicate number (1, 2, 3...). The code connects the 100 µm ID poly(VDM-co-EDMA) trypsin based IMER digest given in **Figure S1** to the morphology of the same poly(VDM-co-EDMA) monolith given in **Section 5 Figure S11**. For the 180 µm ID poly(VDM-co-EDMA) trypsin based IMERs, the digest is given in **Figure S2** and the morphology of the monolith is given in **Section 5 Figure S12**. On-line IMER-LC-MS was demonstrated with one 180 µm ID poly(VDM-co-EDMA) reactor **(Figure S3).** The protein sample consisted of carbonic anhydrase, myoglobin, fibrinogen beta, hemoglobin, cytochrome C, fibrinogen gamma, human serum albumin, fibrinogen alpha and transferrin.


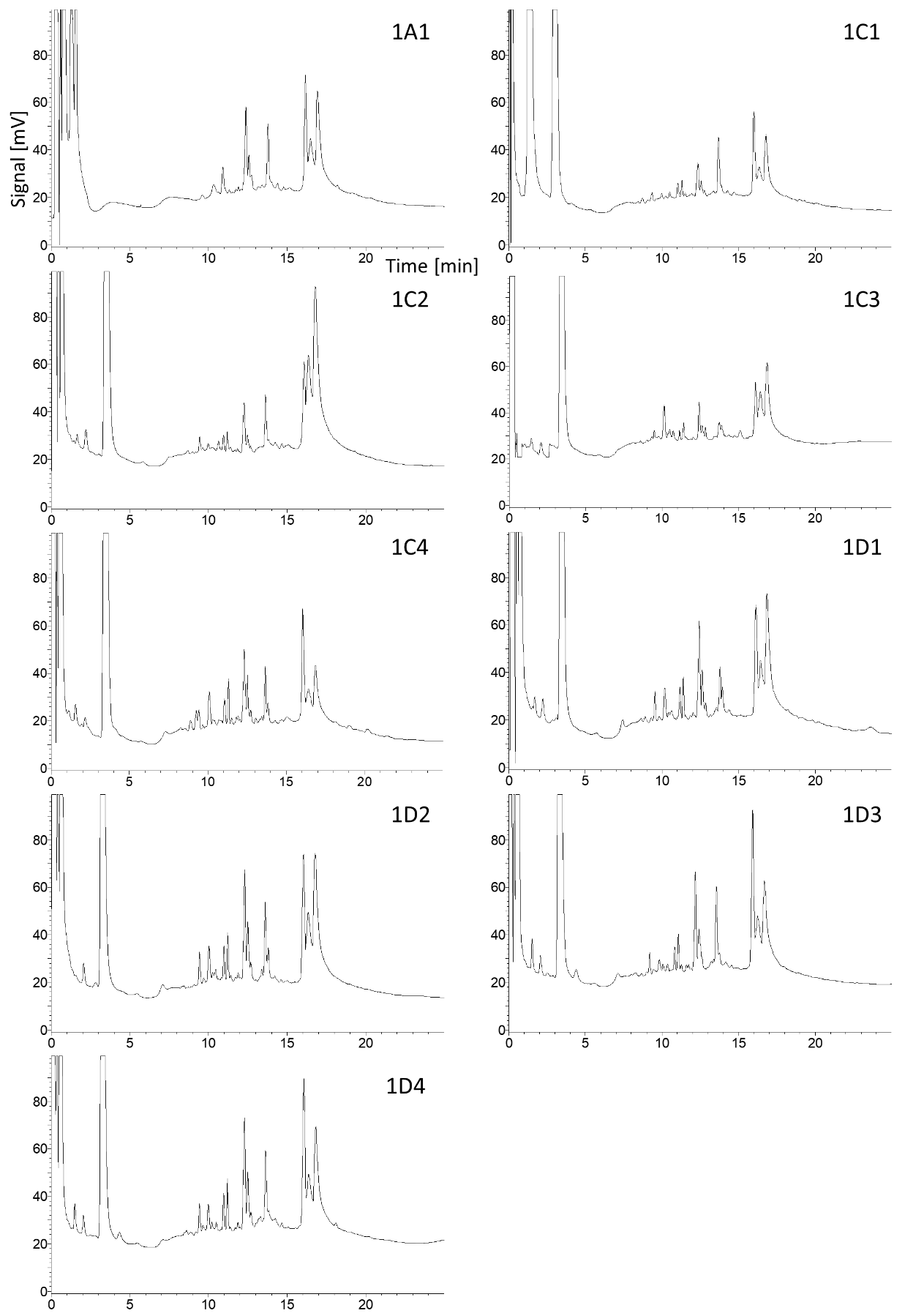
 **Figure S1:** LC-UV chromatograms of 0.6 µL of 500 µg/µL myoglobin digested on 100 µm ID x 160 µm poly(VDM-co-EDMA) trypsin IMER. (n = 9). Chromatographic conditions as described in **Figure 2** in main manuscript. Each chromatogram is marked with a code representing the poly(VDM-co-EDMA) monolith (immobilized with trypsin to digest myoglobin) and the morphology of the monolith is shown in **Section 5** **Figure S11**.


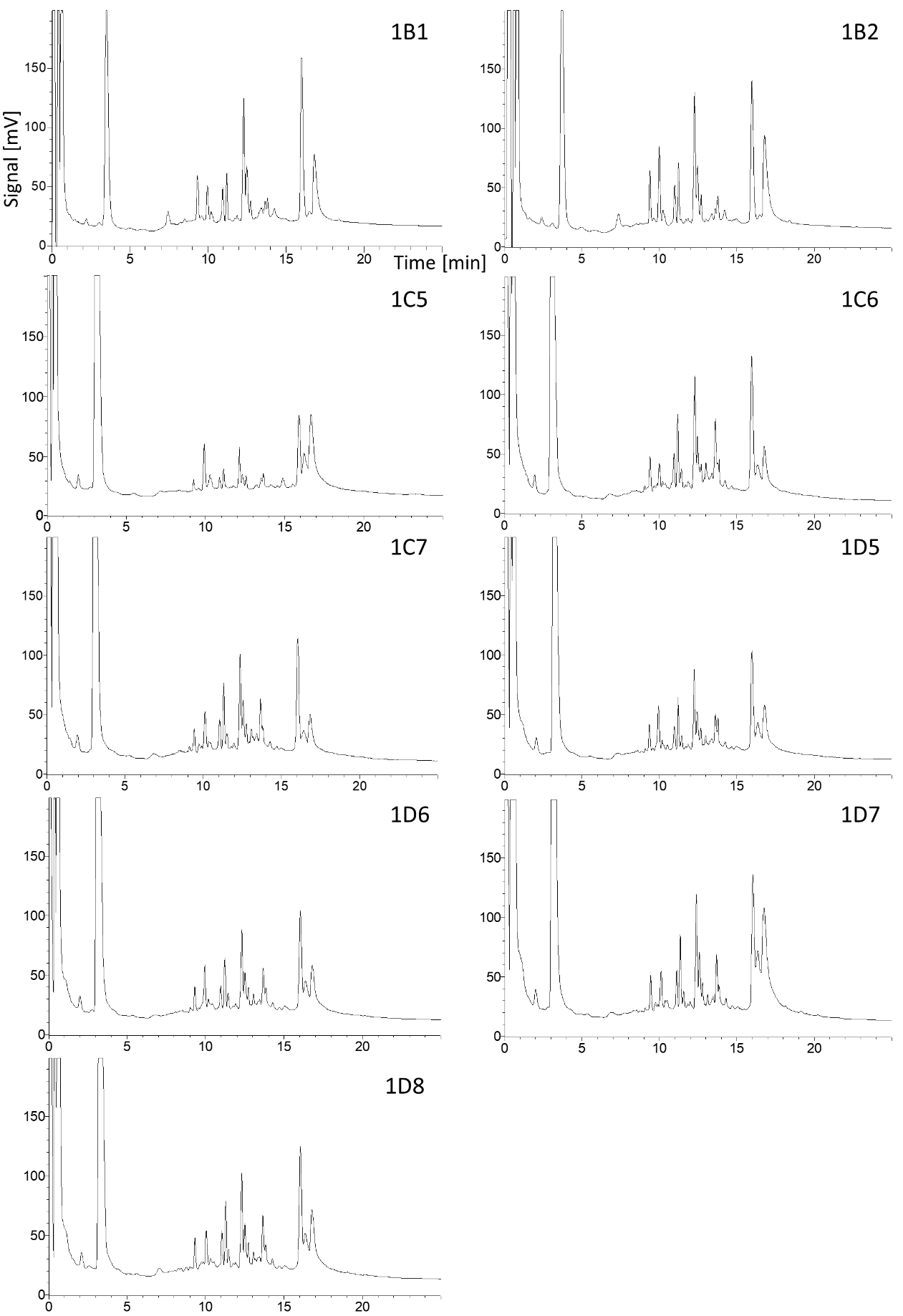

**Figure S2:** LC-UV chromatograms of 1.4 µL of 500 µg/µL myoglobin digested on 180 µm ID x 110 µm poly(VDM-co-EDMA) trypsin IMER. (n = 9). Chromatographic conditions as described in **Figure 2** in main manuscript. Each chromatogram is marked with a code representing the poly(VDM-co-EDMA) monolith (immobilized with trypsin to digest myoglobin) and the morphology of the monolith is shown in **Section 5** **Figure S12**.


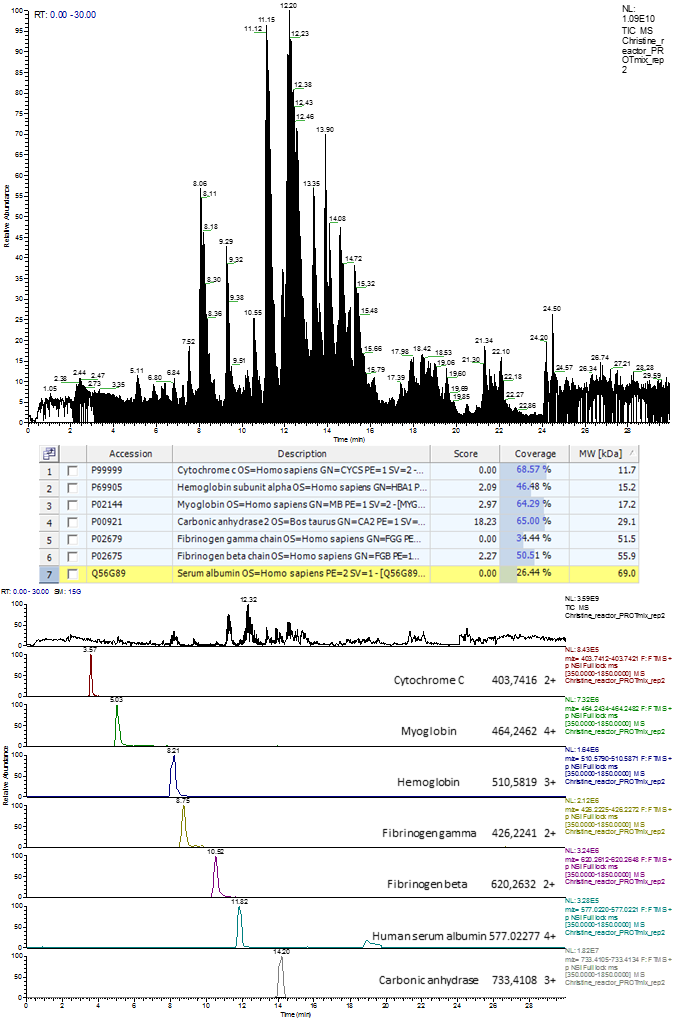

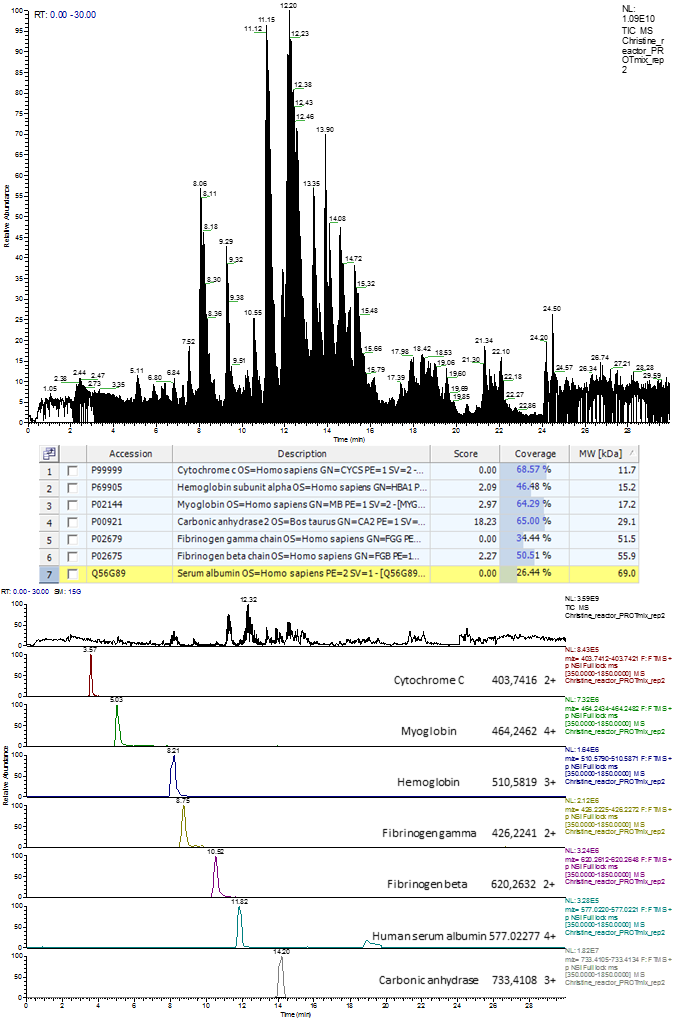


Figure S3: Parameters were as described in [1], with exception of using one IMER reactor (180 µm ID x 110 µm poly(VDM-co-EDMA)) as external sample loop and the pre-column (40 mm x 0.1 mm ID) and analytical column (100 mm x 0.1 mm ID) used in the experiment were silica based monolithic reversed phase columns prepared as described in [2] (similar to Chromolith CapRod C18 capillary columns delivered by Merck Millipore). The gradient was performed with %B: at 5% for 0–2 min, linearly increased to 45% from 2-22 min, quickly increased to 95% for 1 min and kept at 95% at 23–30 min.

# Synthesis of modified LDW639 Wnt-inhibitor

For the synthesis of the modified Wnt-signaling pathway inhibitor LDW639 (**Figure S4**), the following reagents were purchased from Fluorochem (Hadfield, United Kingdom): methyl-4-oxotetrahydro-2H-thiopyran-3-carboxylate (**I**, 95%), 4-boc-aminomethylbenzamidine (**II**, 97%) and 2,2-dimethyl-4-oxo-3,8,11-trioxa-5-azatridecan-13-oic acid (**V**, 97%). In addition, potassium carbonate (anhydrous, Redi-Dri™, ≥ 99%), magnesium sulfate (anhydrous, ReagentPlus, ≥ 99.5%) and N,N-dimethylformamide (DMF, anhydrous, 99.8%) were acquired from Sigma Aldrich (St. Louis, MO, USA). Methanol (MeOH, 100%), ethyl acetate (99.9%) and diethyl ether were purchased from VWR (Radnor, PA, USA). Trifluoroacetic acid (TFA), 4-methylmorpholine (NMM) and 2-(7-aza-1H-benzotriazole-1-yl)-1,1,3,3-tetramethyluronium hexafluorophosphate (HATU) were obtained from Fluorochem. Dichloromethane (DCM, 99.9%) was acquired from Honeywell (Morris Plains, NJ, USA). Acetyl chloride (98%) was obtained through Acros Organics (Geel, Belgium) now a part of Thermo Fisher Scientific (Waltham, MA, USA). Hei-Vap™ value rotavapor from Heidolph (Schwabach, Germany) equipped with a PC 3001 VARIOpro vacuum pump and a CVC 3000 vacuum controller from Vacuubrand (Wertheim, Germany) was used for drying of products. For ^1^H-NMR, DMSO-D^6^ from Euriso-top (Saint Aubin, France) was used as solvent and the spectra were recorded on DPX200 (200 MHz), DPX300 (300 MHz) or AVII400 (400 MHz) NMR instrument from Bruker depending on availability. Standard methods from Bruker were used for recording.


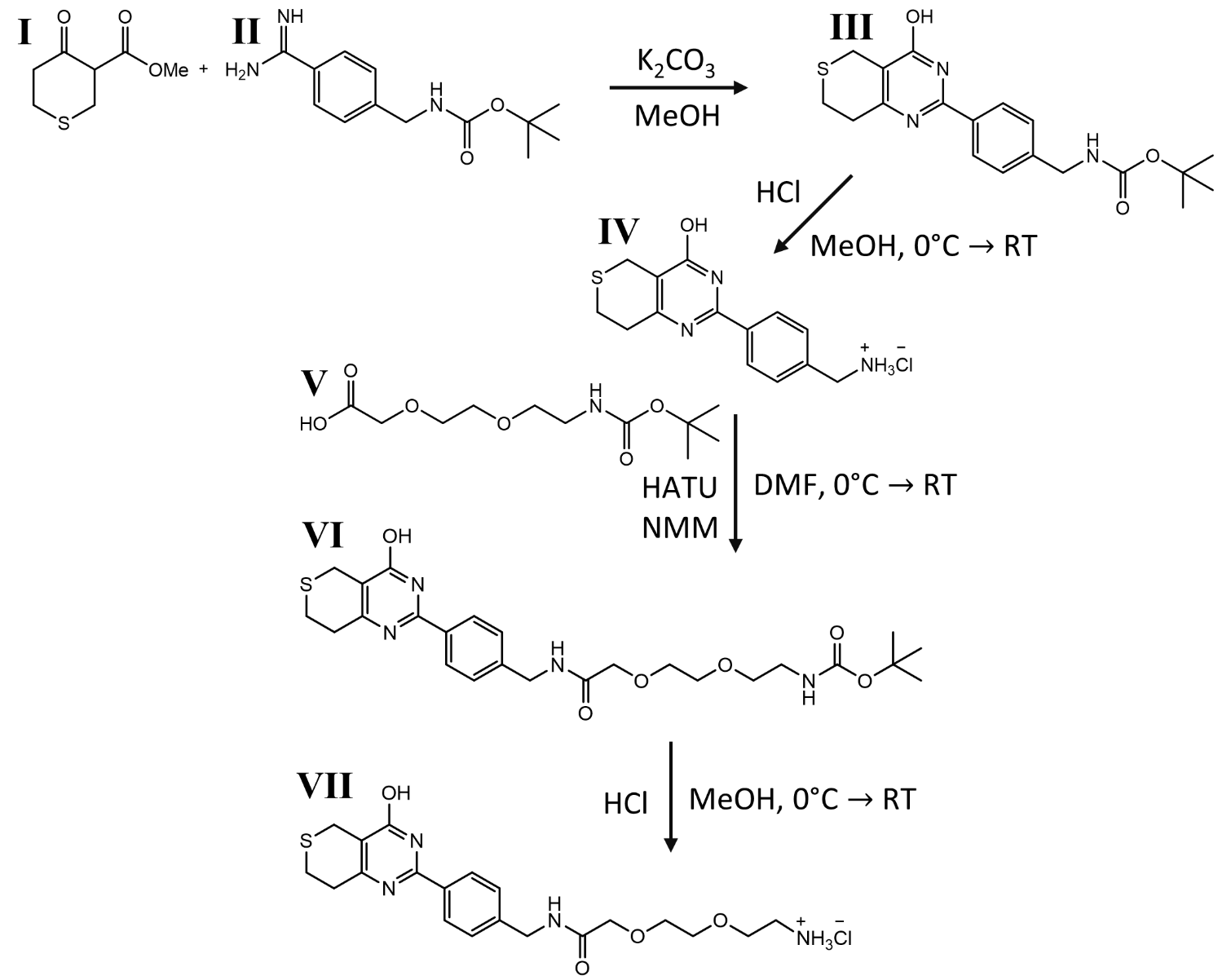


Figure S4: The linear synthesis path for modified LDW639 (VII).

**Step 1: Formation of *tert*-butyloxycarbonyl protected LDW639**

Preparation of *t*-BOC LDW639 (**III**, **Figure S4**) was executed by mixing 1.0 equiv. of **I** with 1.05 equiv. of **II** in MeOH (60 mL for 5 g of reagents). During magnetic stirring 2 equiv. of K_2_CO_3_ was added. After stirring at room temperature (RT) for 18 to 20 hours, the mixture was filtered and washed twice with MeOH before drying. Subsequently, the dried material was dissolved in water (500 mL for 5 g of reagents) and pH adjusted to pH 5 by adding 1 M HCl. The precipitated material was collected by suction filtration and washed with cold water/MeOH (1/1, *v/v*) and cold diethyl ether (75 mL for 5 g of reagents) before drying. The ^1^H-NMR spectra of the crude product (**III**) is shown in **Figure S5**. The procedure was based on Huang *et al.* 2009 [3].


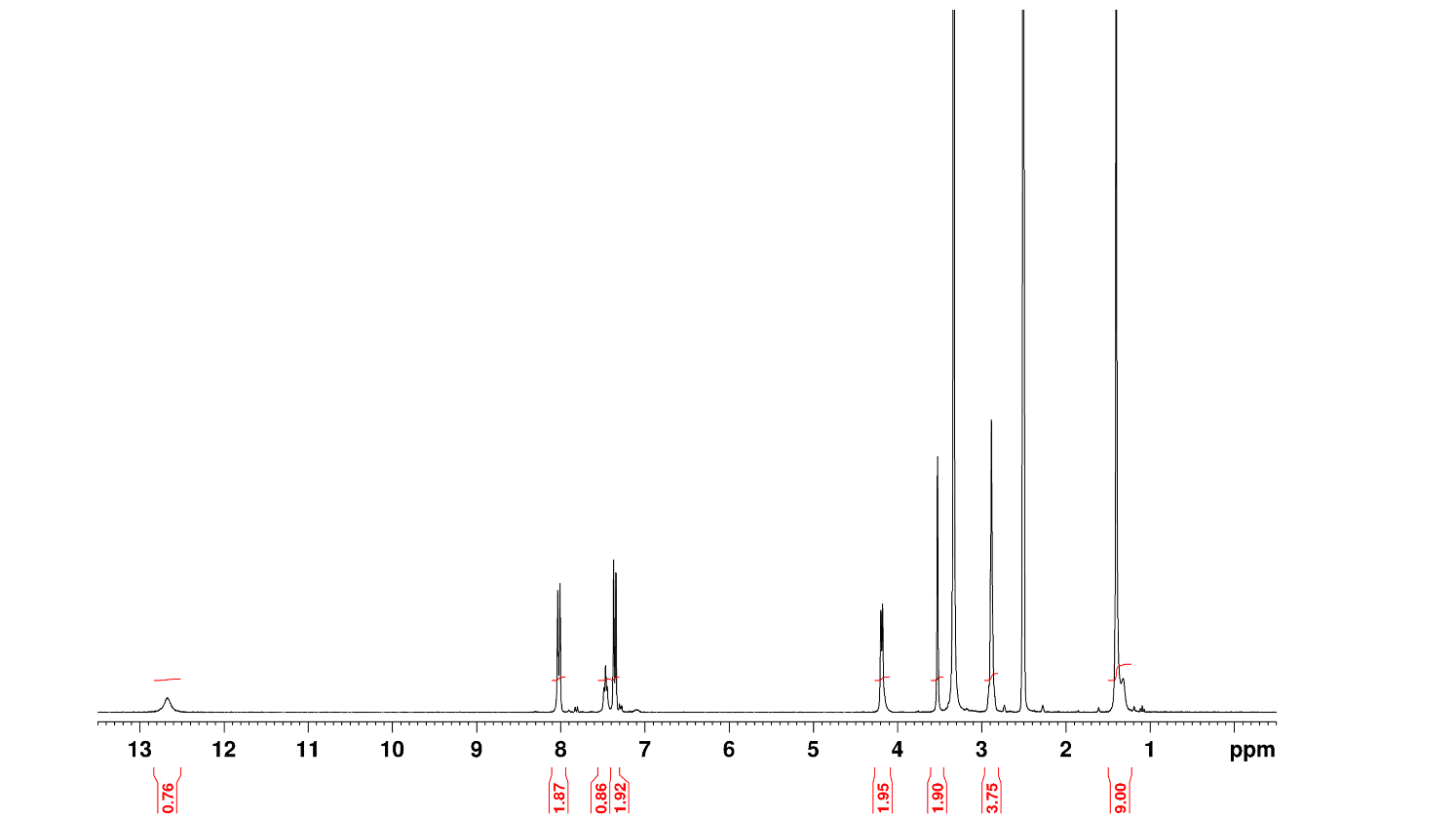


Figure S5: ^1^H-NMR spectrum of *t*-BOC LDW639 (III) dissolved in DMSO-D^6^, 16 scans on DPX300 (300 MHz).

**Step 2: Deprotection of *tert*-butyloxycarbonyl protected LDW639**

The crude product (**III**) was deprotected by dissolving 1.0 equiv of **III** in MeOH (300 mL for 3 g **III**) and cooled to 0 °C prior to gradual addition of 20.0 equiv. of acetyl chloride over 15 min using a cylindrical addition funnel. The mixture was stirred 18 to 20 h, while increasing the temperature from 0 °C to room temperature. After drying, the crude product (**IV**) gave the following ^1^H-NMR spectra (**Figure S6**).


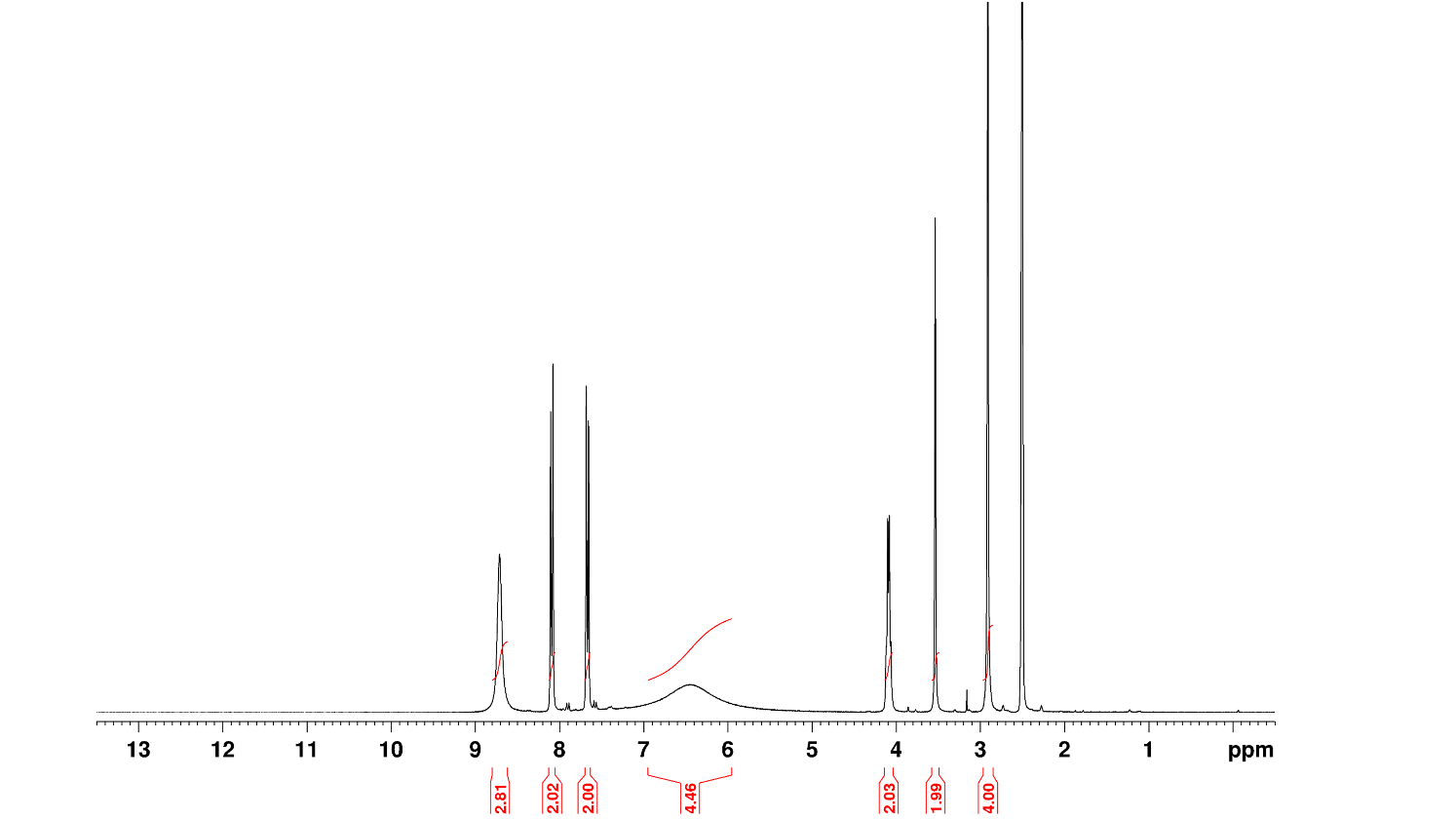


Figure S6: 1H-NMR spectrum of hydrochloric LDW639 salt (IV) dissolved in DMSO-D6, 16 scans on DPX300 (300 MHz).

**Step 3: Adding *tert*-butyloxycarbonyl protected linker through a peptide coupling and product purification by recrystallization**

To a solution of 1.0 equiv. of amine **IV** in DMF (65 mL for 2 g of **IV**) was added 1.05 equiv. of acid **V** and 1.1 equiv. of HATU. After cooling to 0 °C, 2.5 equiv. of NMM was gradually added over 10 min, and the mixture was stirred 18 to 20 h, while increasing the temperature from 0 °C to room temperatur. The crude product was dried and dissolved in 100 mL water and 100 mL saturated K_2_CO_3_ solution. The product was extracted three times with 150 mL ethyl acetate. The pooled organic phases was dried with MgSO_4_ for 60 min prior to filtration, and the solution was dried in vacou offering a powder. The powder was dissolved in 100 mL ethyl acetate by heating under reflux on an oil bath at 70 °C. A hot filtration was executed before the solution was slowly cooled allowing collection of the precipitate by suction filtration and subsequent drying. The purified product **VI** gave the following ^1^H-NMR spectra (**Figure S7**). The intended structure C_25_H_34_N_4_O_6_S was confirmed by ESI-MS at the MS-laboratory at the Department of Chemistry, University of Oslo: with 0.0 ppm deviation from the monoisotopic mass 518.2191 Da.


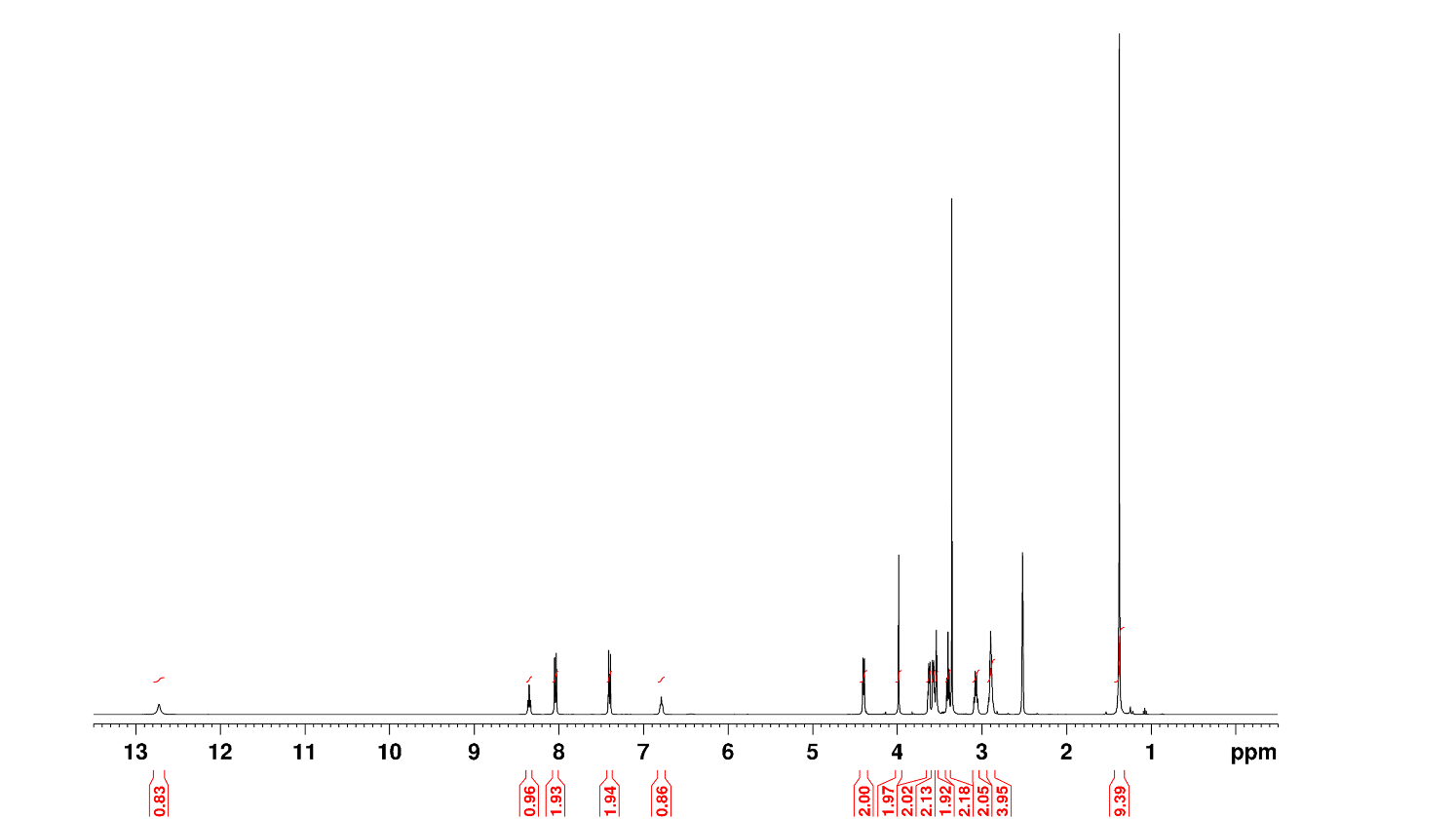


Figure S7: ^1^H-NMR spectrum of *t*-BOC modified LDW639 (VI) dissolved in DMSO-D^6^, 16 scans on DPX300 (300 MHz).

**Step 4: Deprotection to acquire a hydrochloric salt of modified LDW639**

The last step of the synthesis was another deprotection with acetyl chloride. The purified product (**VI**) was deprotected by dissolving 1.0 equiv. of **VI** in MeOH (50 mL for 0.5 g **VI**) and cooled to 0 °C prior to gradual addition of 20.0 equiv. of acetyl chloride over 15 min. Mixture was stirred 18 to 20 h, while increasing the temperature from 0 °C to RT. After drying, the product (**VII**) gave the following ^1^H-NMR spectra (**Figure S8**). The intended structure C_20_H_27_N_4_O_4_S was confirmed by ESI-MS at the MS-laboratory at the Department of Chemistry, University of Oslo: with 0.0 ppm deviation from the monoisotopic mass 419.1748 Da.


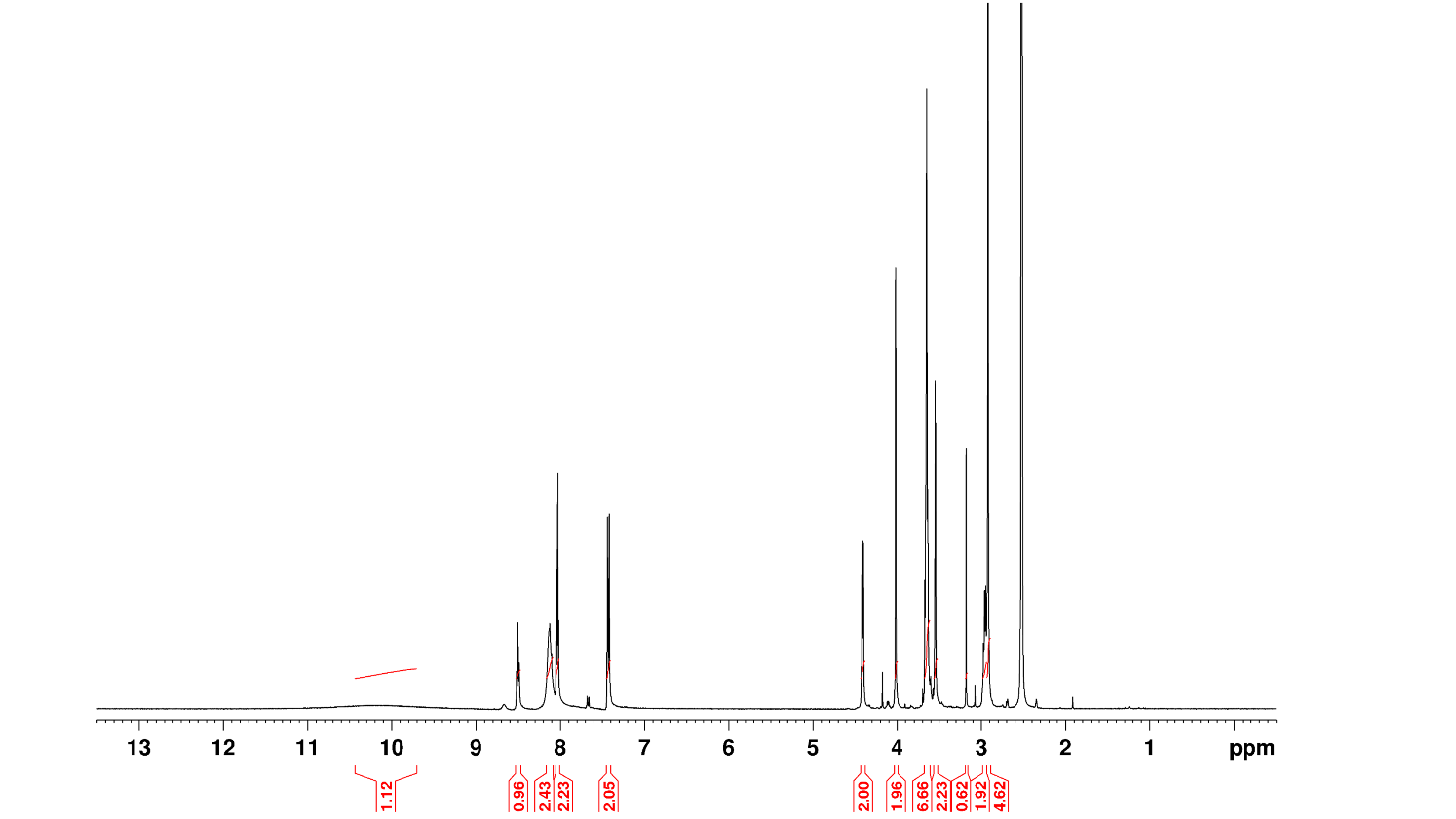


Figure S8: ^1^H-NMR spectrum of modified LDW639 (VII) as hydrochloric salt dissolved in DMSO-D^6^, 16 scans on AVII400 (400 MHz).

**Determination of Wnt-activity**

To establish if the synthesized drug did affect Wnt-signaling, a SuperTOPFlash-luciferase assay (STF-Luc) for Wnt-activity was executed at the Unit of Cell Signaling, Oslo University Hospital. The STF-Luc assay was performed as described by Voronkov *et al.* 2013 [4]. The synthesis product was tested in the following concentration range: 0.12, 0.37, 1.1, 3.3, 10 and 25 μM, and incubated for 24 hours. The activity of both luciferase and *Renilla* was measured. In addition to the synthesis product, HCl controls were analysed in the same concentration range. From plot of % activity of Wnt-signaling pathway vs. concentration of modified LDW639 (**Figure S9A**), the IC_50_-value of modified LDW639 was 10.96 µM. Inhibition of Wnt-signaling pathway by modified LDW639 was not affected by that the drug was added as a hydrochloric salt, since HCl (**Figure S9B**) did not inhibit the Wnt-signaling pathway.


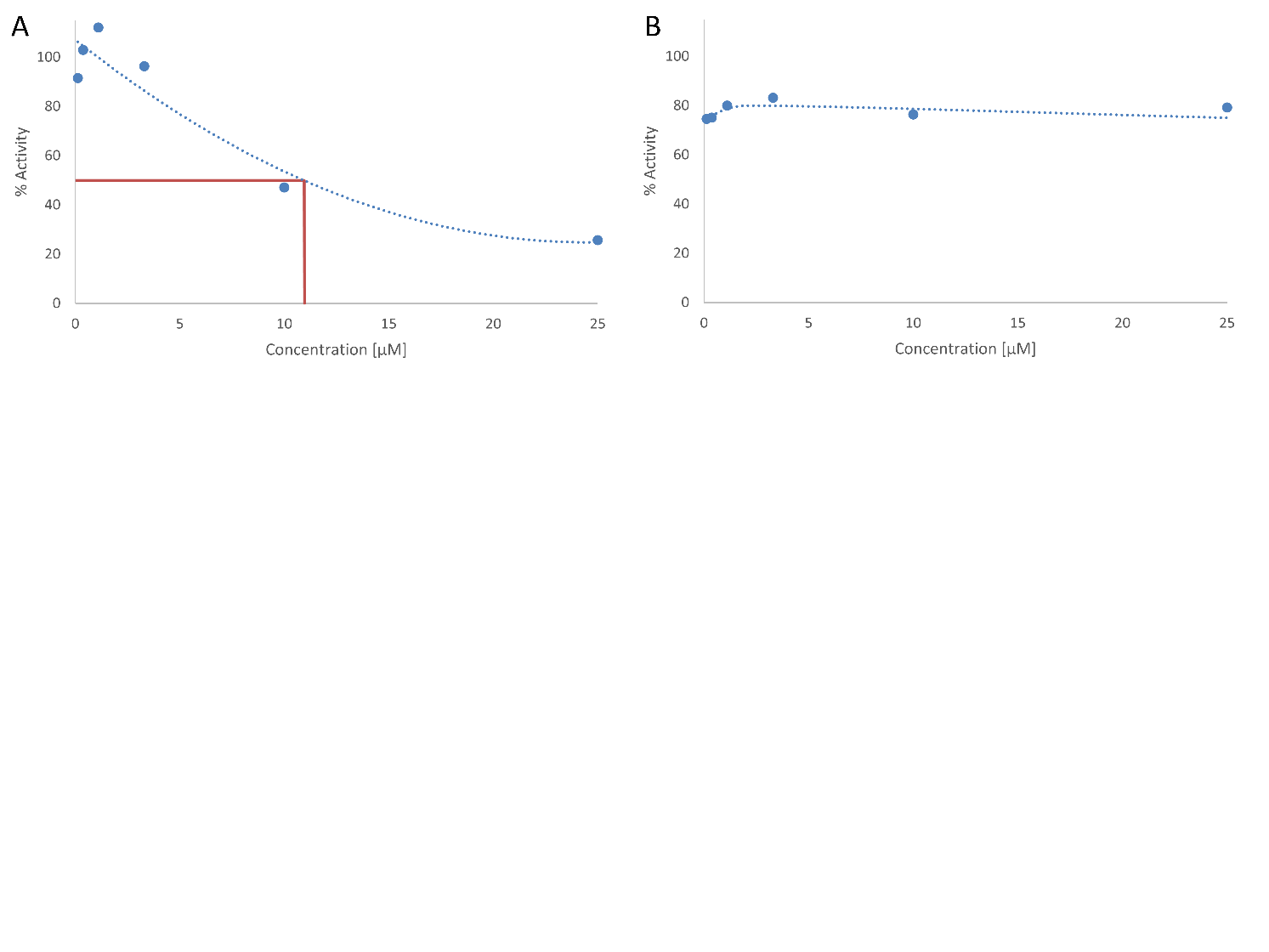


Figure S9: (A) % activity of Wnt-signaling pathway vs. concentration of modified LDW639 showing the inhibition of the Wnt-signaling pathway after 24 hours incubation with modified LDW639. (B) % activity of Wnt-signaling pathway vs. concentration of HCl showing the inhibition of the Wnt-signaling pathway after 24 hours incubation with HCl.

# Western blot analysis for tankyrase 1 and 2, and actin.

Reagents used for western blot; Tris-HCl (pH 6.8), sodium dodecyl sulfate (SDS), glycerol (100%), β-mercaptoethanol (99%), ethylenediaminetetraacetic acid (EDTA), bromophenol blue, trizma® base (≥ 99%) and glycine (≥ 99%) were all from Sigma Aldrich. PageRuler™ Prestained protein ladder was from Thermo Fisher. Tris buffered saline with Tween™ (TBS-T) tablets was purchased from Medicago AB (Uppsala, Sweden). Nonfat dried milk powder was acquired from PanReac AppliChem ITW reagent (Dramstadt, Germany). Technical methanol was acquired from VWR, and radioimmunoprecipitation assay (RIPA) lysis and extraction buffer were obtained from Thermo Fisher Scientific. Amersham™ enhanced chemiluminescence (ECL™)-prime was acquired from GE Healthcare Life Science (Chicago, IL, USA). Primary mouse monoclonal antibody for tankyrase-1/2 (E-10, lot# F0711, cat: sc-365897), horseradish peroxidase (HPR) conjugated polyclonal secondary donkey anti-mouse antibody (cat: sc-2314), HPR conjugated polyclonal secondary donkey anti-rabbit antibody (cat: sc-2313) were acquired from Santa Crus Biotechnology (Dallas, TX, USA). Primary rabbit polyclonal antibody for actin (A2066, lot#018M4753V) was purchased from Sigma Aldrich.

**Gel electrophoresis**

Prior to application of sample on the gel, in-house loading buffer (4x) was mixed 1+3 (*v+v)* with sample diluting it to a 1x loading buffer and heating the samples at 70 °C for 10 min on a heating block from Grant instruments. The in-house loading buffer (4x) consisted of 2.0 mL 1 M tris-HCl (pH 6.8), 0.8 g SDS, 4.0 ml glycerol, 0.4 mL 14.7 M β-mercaptoethanol, 1.0 mL 0.5 M EDTA and 8 mg bromophenol blue. For gel electrophoresis, a 1 mm thick 3-8% tris-acetate Invitrogen NuPAGE™ gel with 10 wells was washed gently with tap water and inserted in a mini Cell electrophoresis chamber, both from Thermo Fisher. The chamber was filled with 25 mL SDS NOVEX® tris-acetate (20X) running buffer, also from Thermo Fisher, diluted to 500 mL with tap water. 20 µL of sample and 10 µL of protein ladder were loaded using microcapillary pipette tips from VWR.

The gel was applied 75 V (200 mA) for 1 hour before the voltage was increased to 110 V (200 mA) for another hour allowing the horizontal bromophenol blue lane to reach the end of the gel. Power supplies were from Bio-Rad (Hercules, CA, USA). After turning off the voltage, the gels were removed from the covers using a spatula. The gels were cut at the bromophenol blue line, and the wells were cut away. The gels were rinsed and incubated with an in-house transfer buffer (1x) for 15 min. The in-house 10x transfer buffer was made by mixing 30.3 g Trizma® base and 144.0 g glycine, and diluting to 1000 mL with water. The 1x transfer buffer was made by mixing 100 mL of the 10x buffer with 200 mL technical methanol and 700 mL water.

**Immunoblotting**

A 45 μm nitrocellulose membrane from Thermo Scientific was cut to match the size of the gels for protein transfer. The membrane was washed in transfer buffer (1x) for 15 min. Protein transfer from gel to membrane was done building a “transfer sandwich” in the following order in the cell: filter paper, membrane, gel and filter paper in a Trans-Blot® SD semi-dry transfer cell from Bio-Rad. The filter paper (extra thick Blot Filter paper from Bio-Rad) was partly soaked in transfer buffer (1x). Each layer was rolled with a 10 mL pipette to remove air bubbles and kept semi-dry by adding transfer buffer (1x). The chamber was left overnight at 4 °C with 1 W applied.

On the following day, the membranes were cut according to the size of the gels. A TBS-T solution was made by dissolving 10 TBS-T tablets in 5 L water. Milk solution was made by dissolving 10 g nonfat dried milk powder in 200 mL TBS-T, yielding a 5% milk in TBS-T (w/v). The membranes were then blocked for 1 hour in 5% nonfat-milk in TBS-T on a mixing plate from Edmund Bühler GmbH (Bodelshausen, Germany). To 5 mL of 5 % nonfat-milk in TBS-T in a 50 mL tube, 20 μL of primary antibody for tankyrase-1/2 (host animal mouse) was added (1:250 dilution). The membranes were transferred to the tubes with primary antibody and incubated at 4 °C on a roller from Stuart (Stone Straffordshire, UK) overnight.

Before incubation with secondary antibody (donkey-antimouse, diluted 1:5000 in 5 % nonfat-milk in TBS-T), the membranes were rinsed and washed in TBS-T on a mixing plate for 20 min. Transferring the membrane to tubes containing secondary antibodies in a solution, the membranes were incubated with secondary antibodies for 2 hours on a roller at room temperature.

**Visualization**

The membranes were twice rinsed and washed with TBS-T for 20 min on a mixing plate. Enhanced chemilumiscence prime (ECL-prime) was made by mixing the two components 1+1 *(v+v)* giving 500 μL per membrane. The ECL-prime was pipetted back and forth over the membranes for 3 min. Finally, the membranes were placed between two sheets of Nobo transparency film obtained from ACCO Brands (Lake Zurich, IL, US) and covered with additional ECL-prime. Bubbles were removed by stroking filter paper over the film surface. The protein bands were developed with Chemidoc touch imaging system from Bio-Rad and the raw files were treated using Image Lab version 6.0.1 also from Bio-Rad.

**Loading control**

After visualization the membranes were twice rinsed and washed with TBS-T for 20 min on a mixing plate, before primary (rabbit-antiactin, dilution 1:1000 in 5% nonfat-milk in TBS-T) and secondary (donkey-antirabbit, dilution 1:5000 in 5% nonfat-milk in TBS-T) antibodies for actin were applied in the same manner as described above and the membranes were visualized again for actin.

Raw files from visualization of TNKS1/2 and actin in eluates collected from a CRAM reactor and a MEA monolith are presented in **Figure S10**.


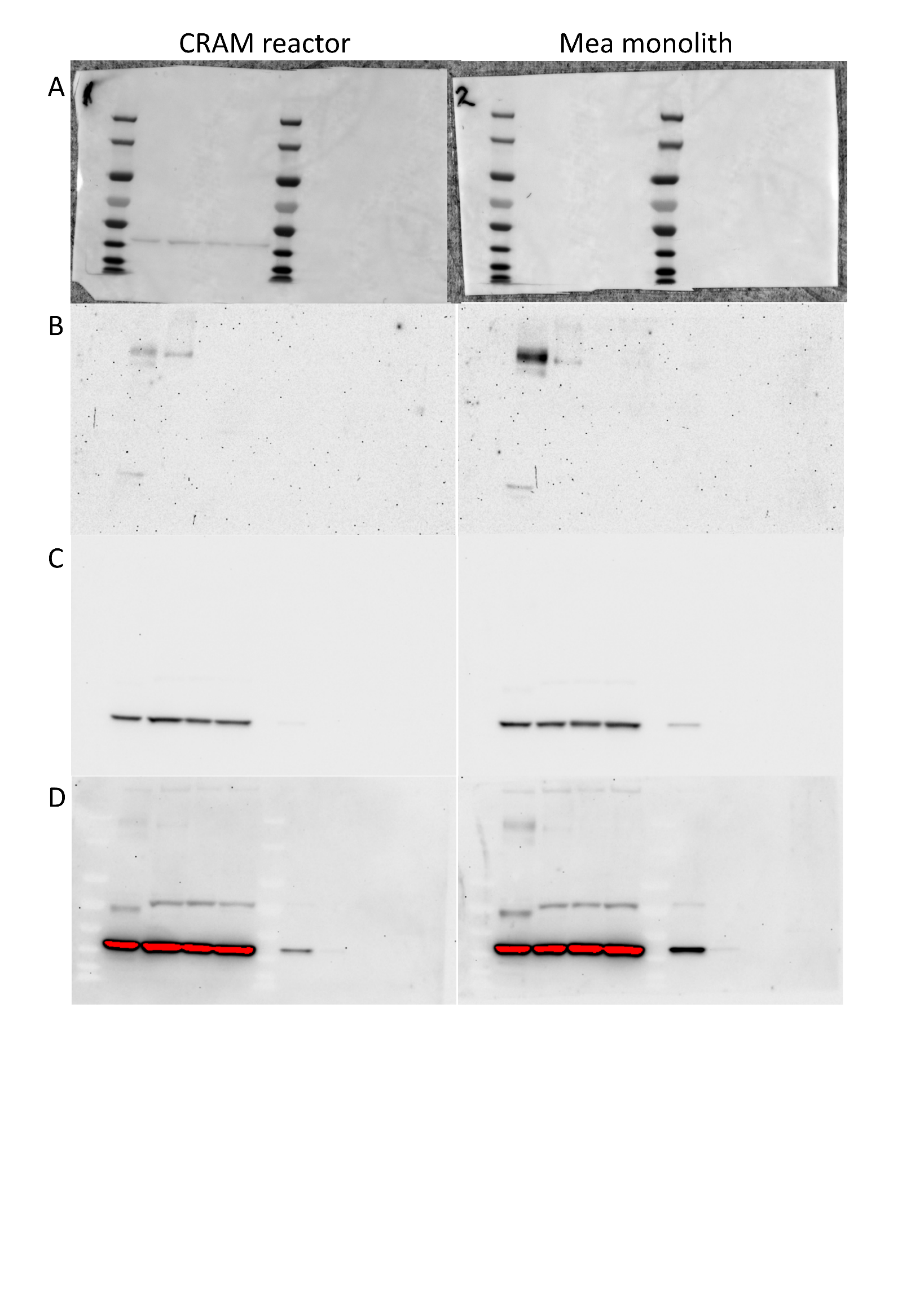


Figure S10: Western blots of: (A) Protein ladder run on a 3-8% tris acetate gel with tris acetate running buffer, from the top the bars marks 150, 120, 85, 65, 40, 30, 25 kDa. (B) TNKS1/2, 7200 s exposure time. (C) Actin, 10 s exposure time. (D) Actin, 180 s exposure time.

# Characterization of morphology of poly(VDM-co-EDMA) monoliths by scanning electron microscopy

The micrographs of the cross-section of the 100 µm ID and 180 µm ID poly(VDM-co-EDMA) monoliths prepared for IMER evaluation are presented in **Figure S11** and **Figure S12**, respectively. Following in **Figure S13** and **Figure S14** are the micrographs of the cross-section of the 180 µm ID and 250 µm ID poly(VDM-co-EDMA) monoliths prepared for CRAM reactor evaluation. The monoliths prepared with the same batch of polymerization solution have an identical number and letter in the beginning of the labels (e.g. 1C1 and 1C5), the last number is the replicate number.


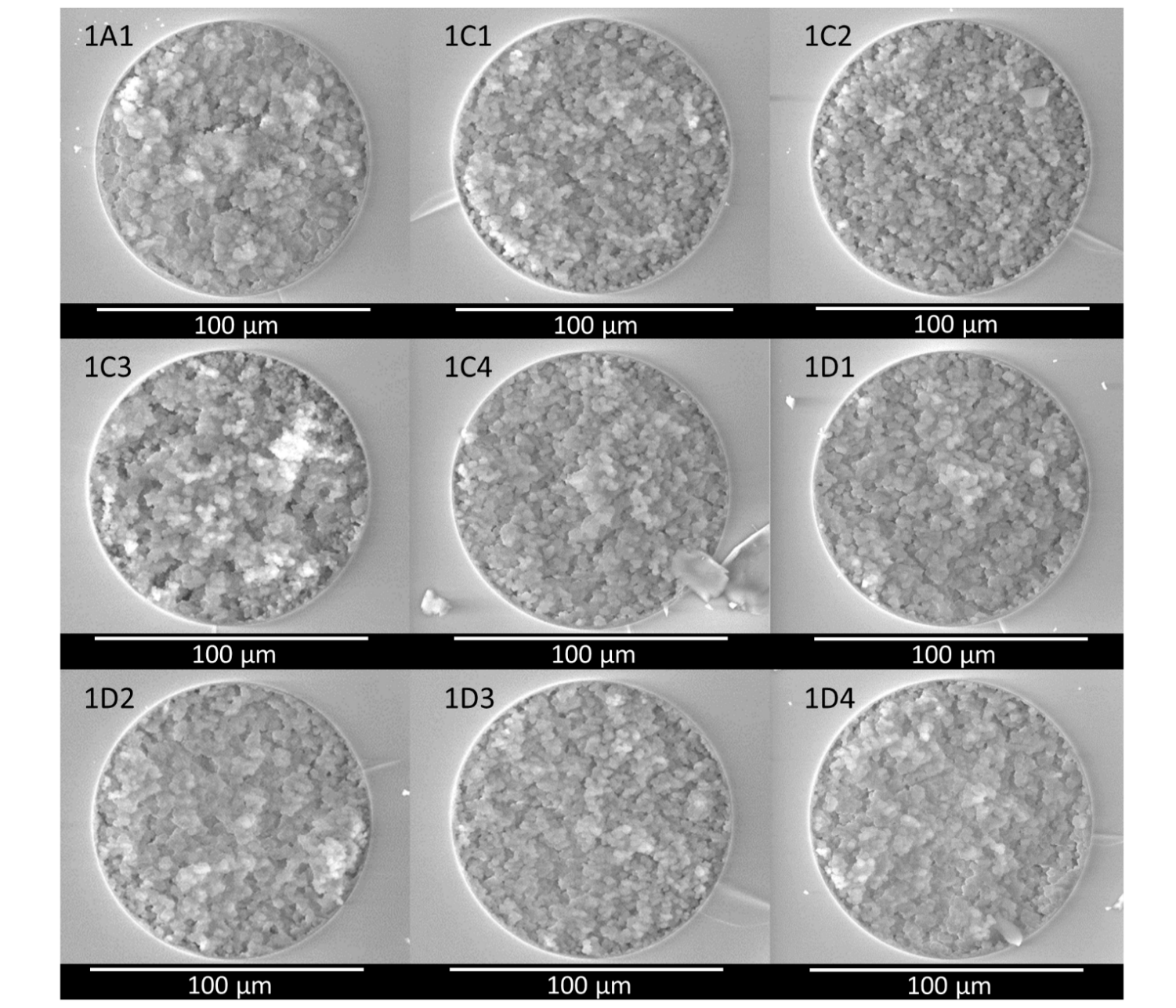
**Figure S11:** Micrographs captured by scanning electron microscopy of 9 replicates of 100 µm ID poly(VDM-co-EDMA) monoliths. The poly(VDM-co-EDMA) monoliths are marked with a code representing: Which project (1=IMER or 2=CRAM), which polymerization solution batch (A, B, C...), and replicate number (1, 2, 3...). The digest executed on the monolith is given **Figure S1**.


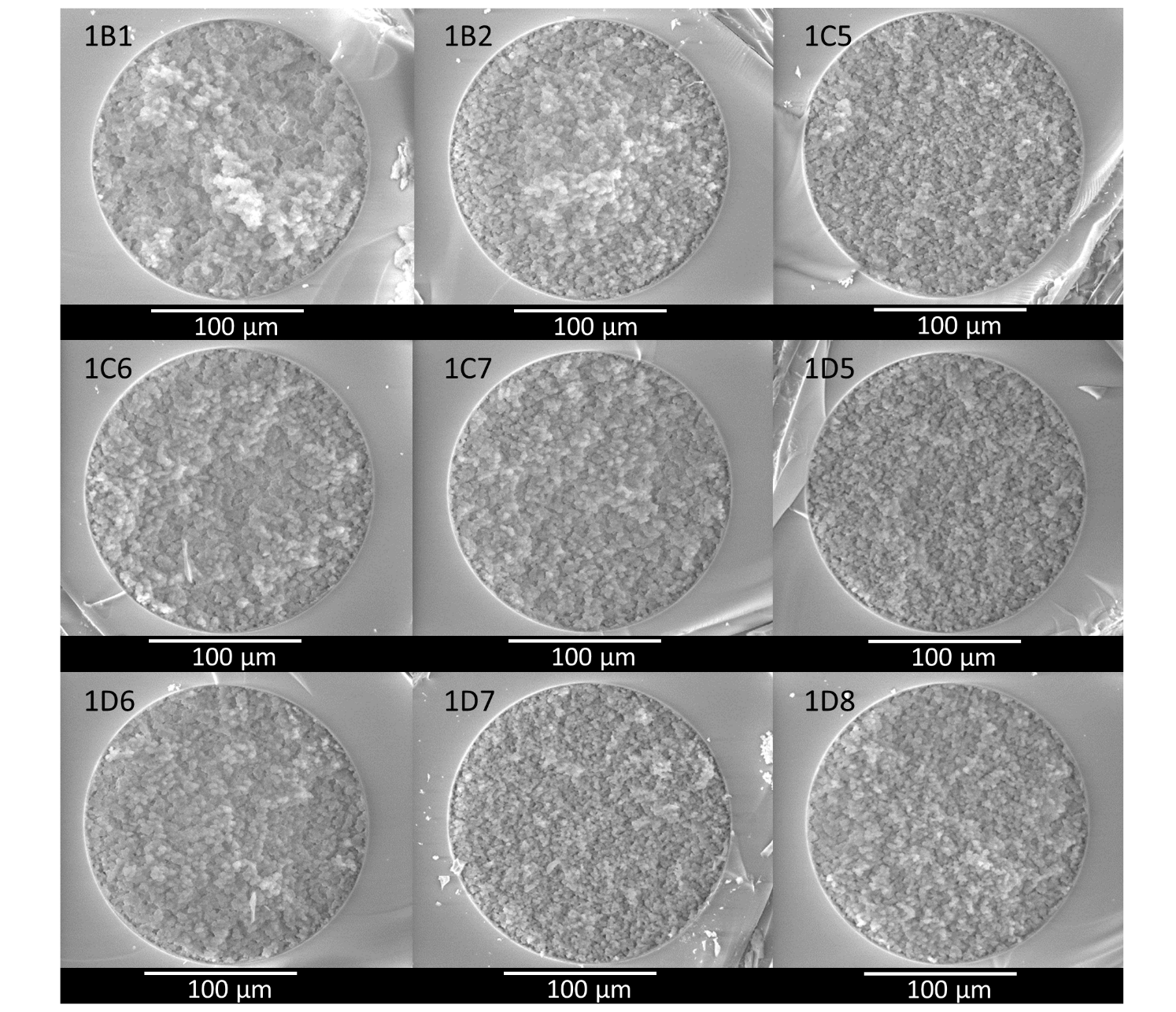


Figure S12: Micrographs captured by scanning electron microscopy of 9 replicates of 180 µm ID poly(VDM-co-EDMA) monoliths. The poly(VDM-co-EDMA) monoliths are marked with a code representing: Which project (1=IMER or 2=CRAM), which polymerization solution batch (A, B, C...), and replicate number (1, 2, 3...). The digest executed on the monolith is given Figure S2.


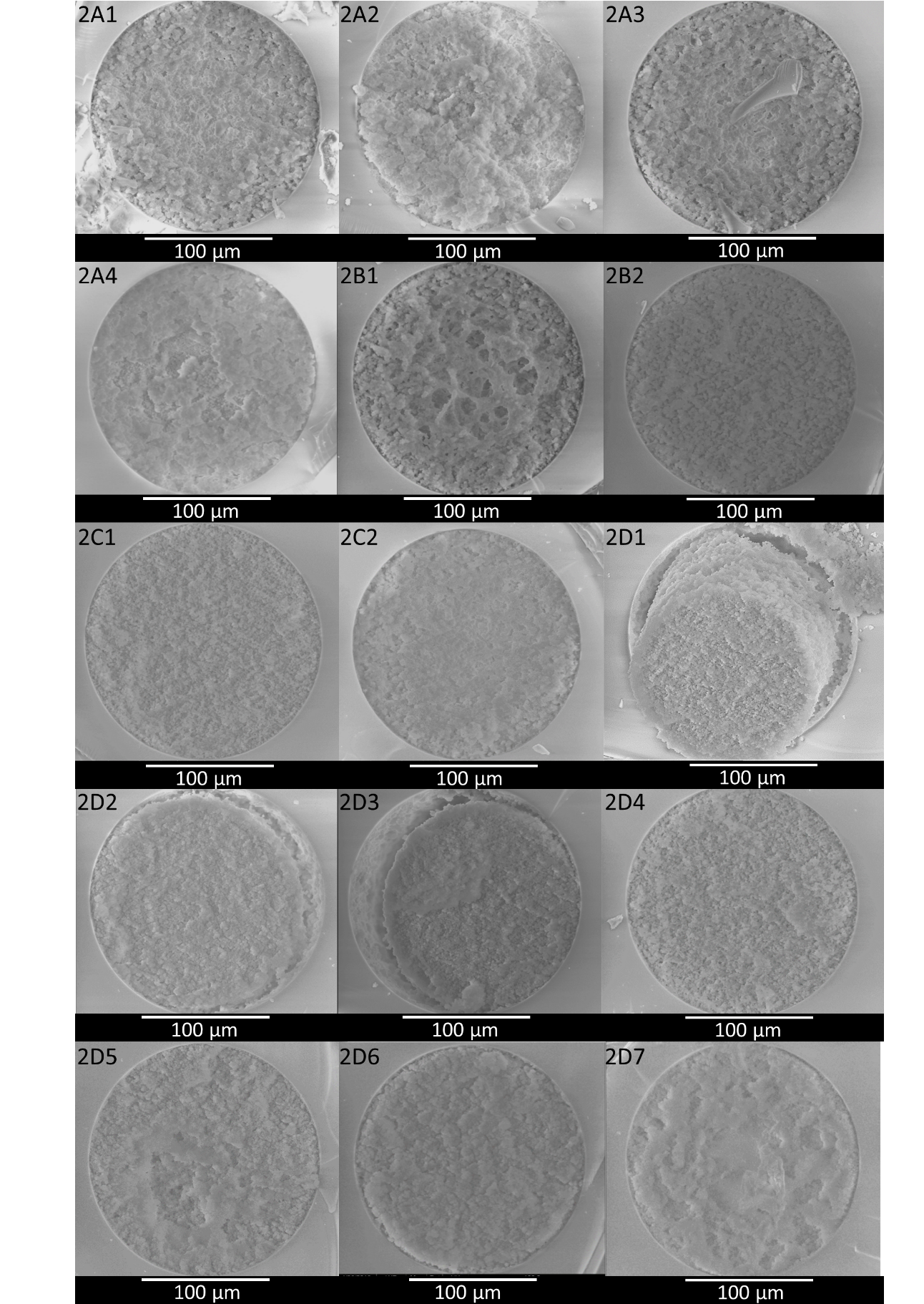

**Figure S13**: Micrographs captured by scanning electron microscopy of 15 replicates of 180 µm ID poly(VDM-co-EDMA) monoliths. The poly(VDM-co-EDMA) monoliths are marked with a code representing: Which project (1=IMER or 2=CRAM), which polymerization solution batch (A, B, C...), and replicate number (1, 2, 3...).


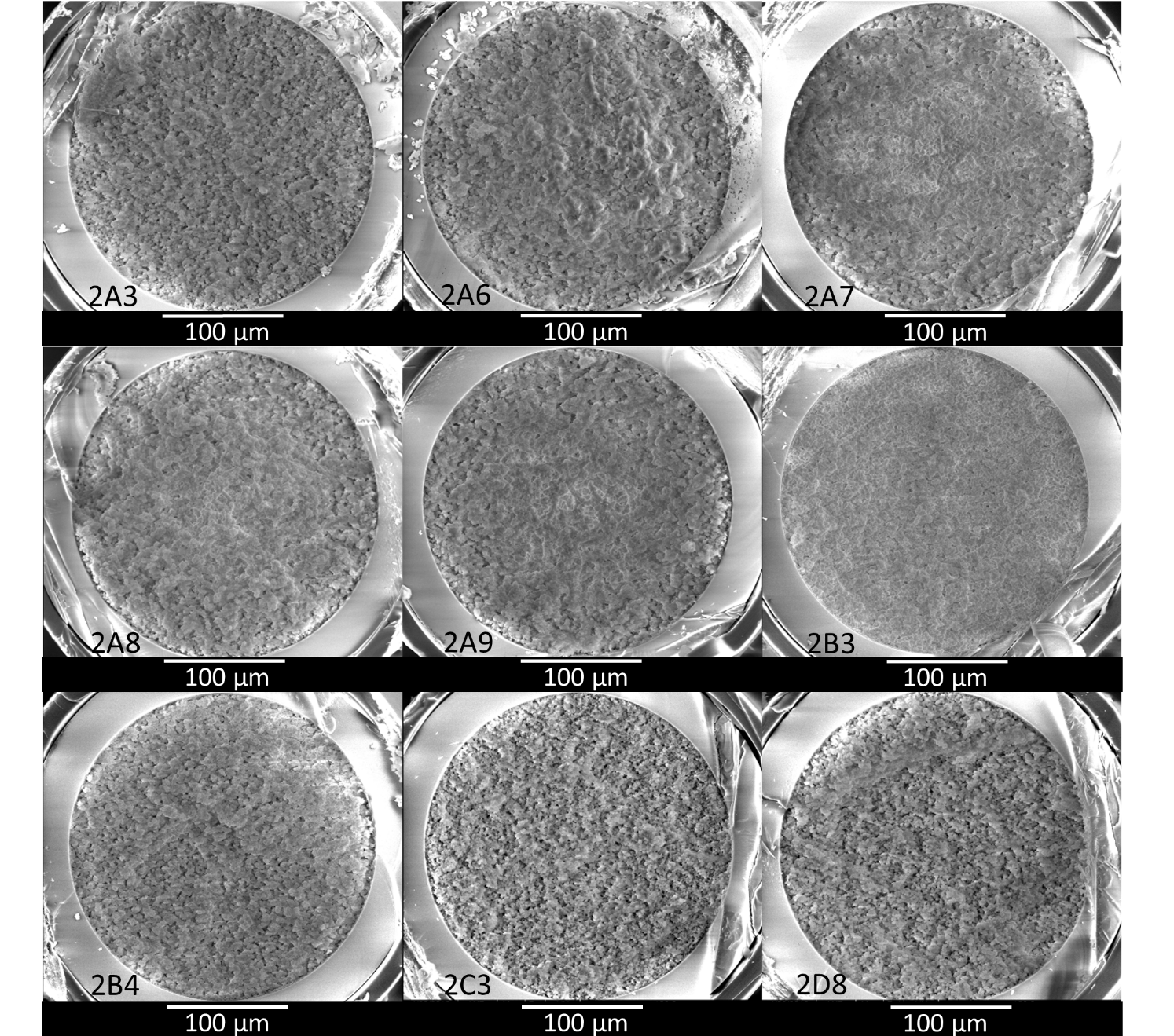


**Figure S14**: Micrographs captured by scanning electron microscopy of 9 replicates of 250 µm ID poly(VDM-co-EDMA) monoliths. The poly(VDM-co-EDMA) monoliths are marked with a code representing: Which project (1=IMER or 2=CRAM), which polymerization solution batch (A, B, C...), and replicate number (1, 2, 3...).

# Proton nuclear magnetic resonance spectra of chemicals used in monolith production

The liquid chemicals (*i.e.* DMF, γ-MAPS, VDM, EDMA, 1-propanol and 1,4-butanediol) were replaced during the study and both the new and old chemicals were analyzed by standard proton nuclear magnetic resonance (1H-NMR) methods (**Figure S15** to **Figure S20**). The spectra were recorded on an AVII400 NMR instrument (400 MHz) with a BACS-120 automatic sample changer both from Bruker (Billerica, MA, USA) and the chemicals were dissolved 1+9 in deuterated dimethyl sulfoxide (DMSO-D^6^) from Cambridge Isotope Laboratories (Tewksbury, MA, USA).


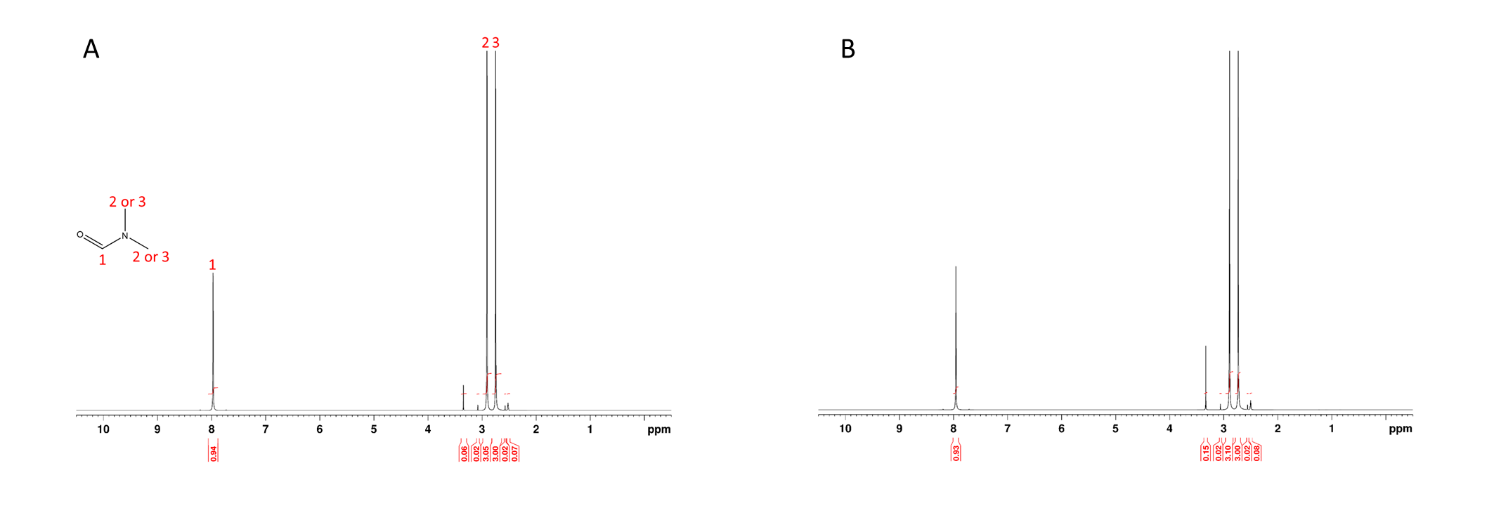


**Figure S15**: ^1^H-NMR spectra of DMF dissolved 1+9 in DMSO-D6, 16 scans on AVII400 (400 MHz): (**A**) “new” and (**B**) “old”**.**


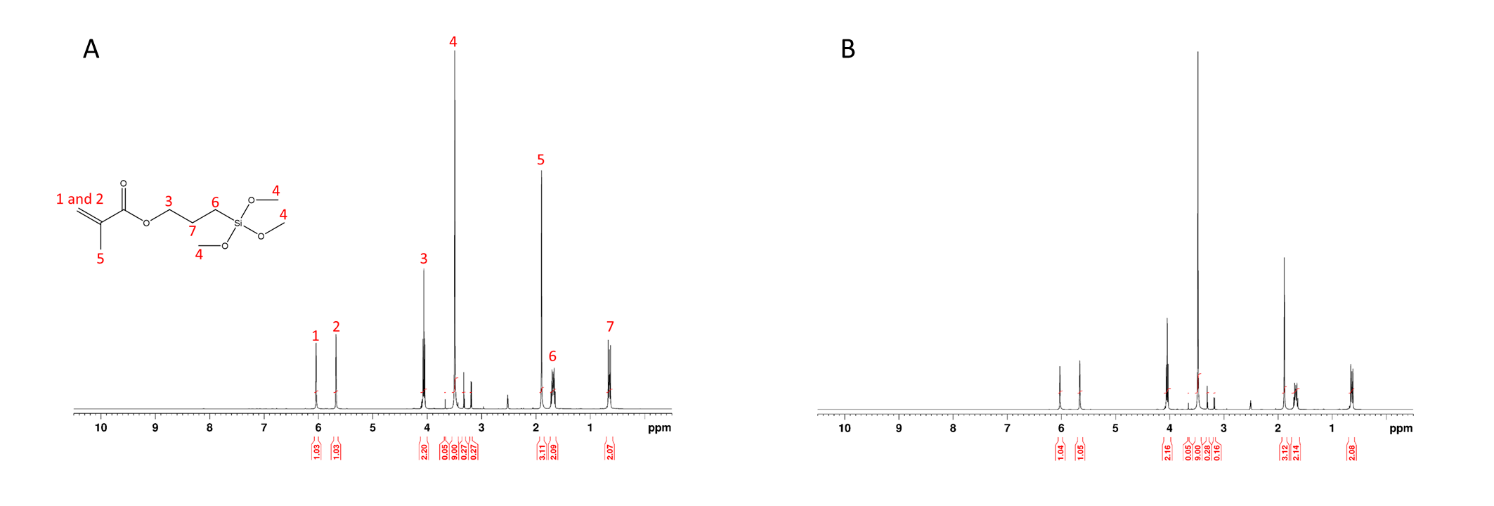


**Figure S16**: ^1^H-NMR spectra of γ-MAPS dissolved 1+9 in DMSO-D6, 16 scans on AVII400 (400 MHz): (**A**) “new” and (**B**) “old”**.**


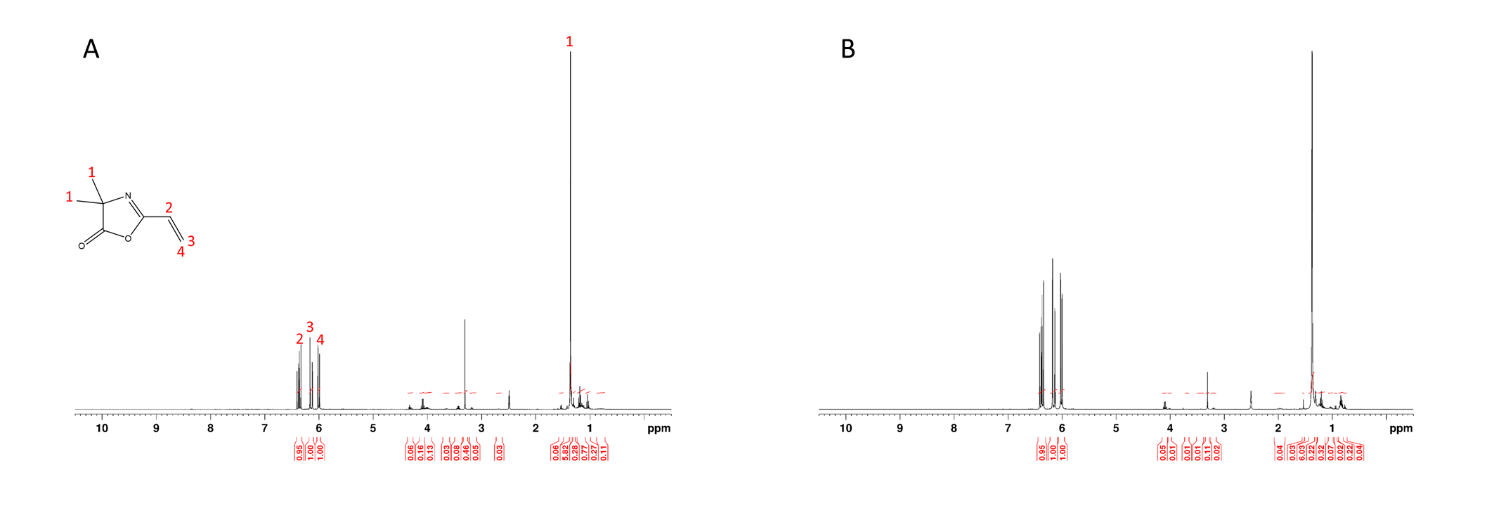


Figure S17: ^1^H-NMR spectra of VDM dissolved 1+9 in DMSO-D6, 16 scans on AVII400 (400 MHz): (A) “new” and (B) “old”.


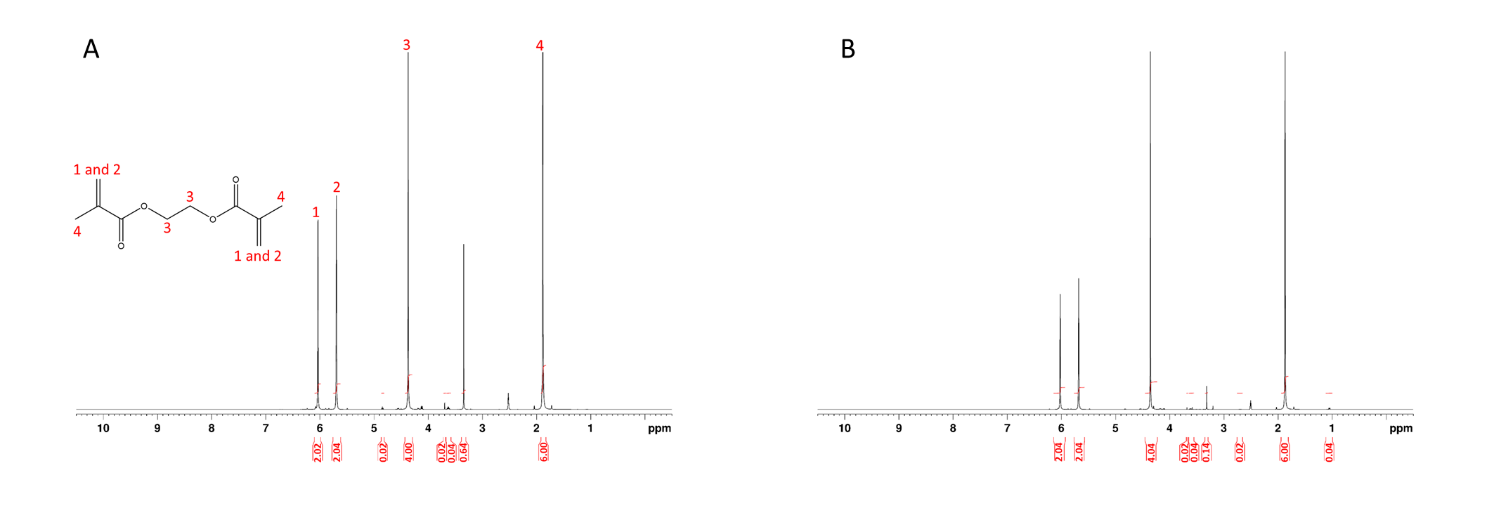


Figure S18: ^1^H-NMR spectra of EDMA dissolved 1+9 in DMSO-D6, 16 scans on AVII400 (400 MHz): (A) “new” and (B) “old”.


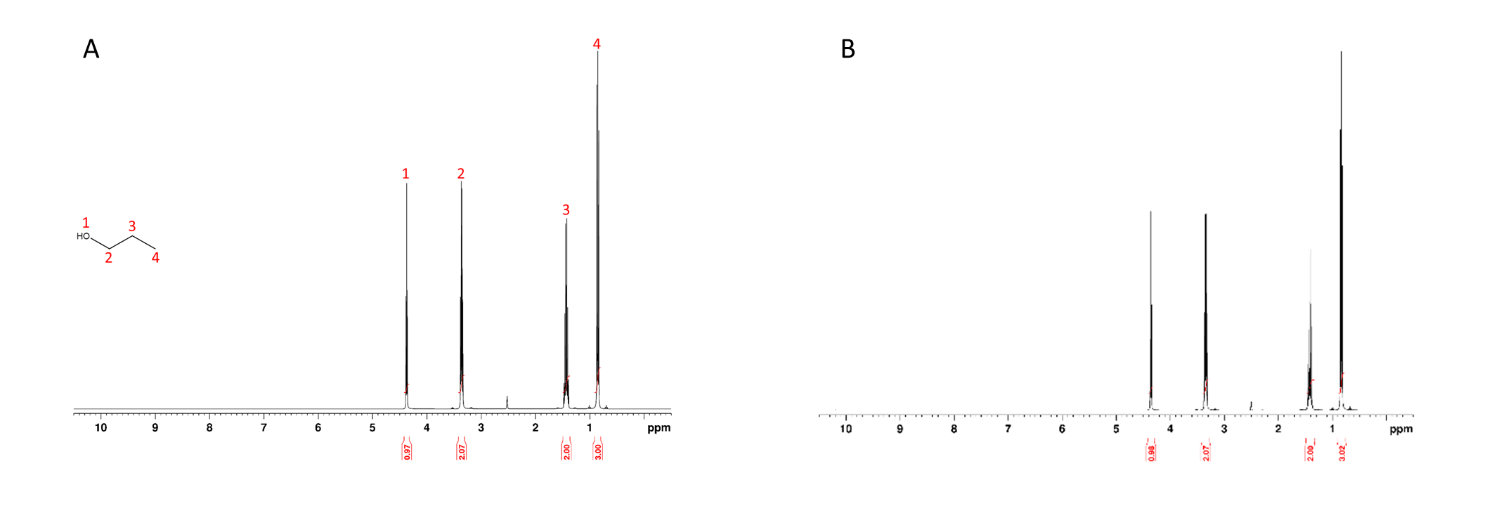
**Figure S19**: ^1^H-NMR spectra of 1-propanol dissolved 1+9 in DMSO-D6, 16 scans on AVII400 (400 MHz): (A) “new” and (B) “old”.


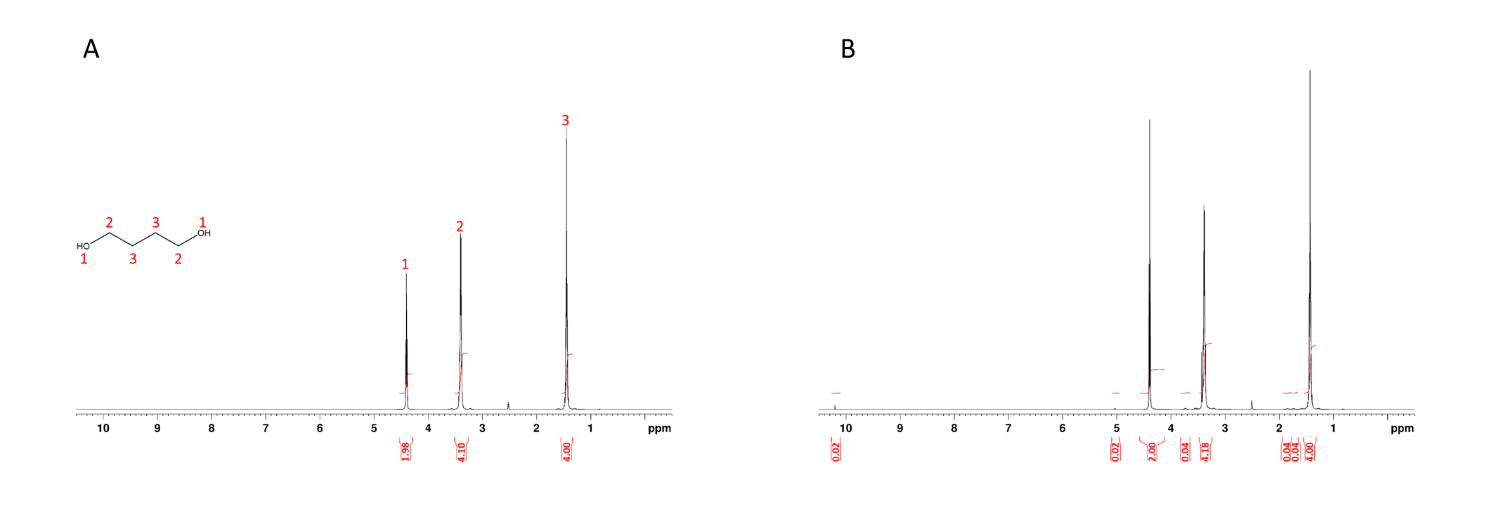


Figure S20: ^1^H-NMR spectra of 1,4-butanediol dissolved 1+9 in DMSO-D6, 16 scans on AVII400 (400 MHz): (A) “new” and (B) “old”.

# References

[1] H.S. Berg, K.E. Seterdal, T. Smetop, R. Rozenvalds, O.K. Brandtzaeg, T. Vehus, E. Lundanes, S.R. Wilson, Self-packed core shell nano liquid chromatography columns and silica-based monolithic trap columns for targeted proteomics, Journal of Chromatography A, 1498 (2017) 111-119.

[2] K. Miyamoto, T. Hara, H. Kobayashi, H. Morisaka, D. Tokuda, K. Horie, K. Koduki, S. Makino, O. Núñez, C. Yang, High-efficiency liquid chromatographic separation utilizing long monolithic silica capillary columns, Analytical chemistry, 80 (2008) 8741-8750.

[3] S.-M.A. Huang, Y.M. Mishina, S. Liu, A. Cheung, F. Stegmeier, G.A. Michaud, O. Charlat, E. Wiellette, Y. Zhang, S. Wiessner, Tankyrase inhibition stabilizes axin and antagonizes Wnt signalling, Nature, 461 (2009) 614-620.

[4] A. Voronkov, D.D. Holsworth, J. Waaler, S.R. Wilson, B. Ekblad, H. Perdreau-Dahl, H. Dinh, G. Drewes, C. Hopf, J.P. Morth, Structural basis and SAR for G007-LK, a lead stage 1, 2, 4-triazole based specific tankyrase 1/2 inhibitor, Journal of Medicinal Chemistry, 56 (2013) 3012-3023.
